# Synthesis and Biological Evaluation of *O*^6^-Aminoalkyl-Hispidol Analogs as Multifunctional Monoamine Oxidase-B Inhibitors towards Management of Neurodegenerative Diseases

**DOI:** 10.3390/antiox12051033

**Published:** 2023-04-29

**Authors:** Ahmed H. E. Hassan, Hyeon Jeong Kim, Keontae Park, Yeonwoo Choi, Suyeon Moon, Chae Hyeon Lee, Yeon Ju Kim, Soo Bin Cho, Min Sung Gee, Danbi Lee, Jong-Hyun Park, Jong Kil Lee, Jong Hoon Ryu, Ki Duk Park, Yong Sup Lee

**Affiliations:** 1 Department of Medicinal Chemistry, Faculty of Pharmacy, Mansoura University, Mansoura 35516, Egypt; 2 Medicinal Chemistry Laboratory, Department of Pharmacy, College of Pharmacy, Kyung Hee University, Seoul 02447, Republic of Korea; 3 Convergence Research Center for Diagnosis, Treatment and Care System of Dementia, Korea Institute of Science and Technology (KIST), Seoul 02792, Republic of Korea; 4 Department of Biomedical and Pharmaceutical Sciences, Kyung Hee University, 26 Kyungheedae-ro, Seoul 02447, Republic of Korea; 5 Department of Fundamental Pharmaceutical Science, Graduate School, Kyung Hee University, 26 Kyungheedae-ro, Seoul 02447, Republic of Korea; 6 Department of Oriental Pharmaceutical Science College of Pharmacy, Kyung Hee University, Seoul 02447, Republic of Korea

**Keywords:** multifunctional molecules, natural products analogs, MAO-B inhibitors, neuroinflammation

## Abstract

Oxidative catabolism of monoamine neurotransmitters by monoamine oxidases (MAOs) produces reactive oxygen species (ROS), which contributes to neuronal cells’ death and also lowers monoamine neurotransmitter levels. In addition, acetylcholinesterase activity and neuroinflammation are involved in neurodegenerative diseases. Herein, we aim to achieve a multifunctional agent that inhibits the oxidative catabolism of monoamine neurotransmitters and, hence, the detrimental production of ROS while enhancing neurotransmitter levels. Such a multifunctional agent might also inhibit acetylcholinesterase and neuroinflammation. To meet this end goal, a series of aminoalkyl derivatives of analogs of the natural product hispidol were designed, synthesized, and evaluated against both monoamine oxidase-A (MAO-A) and monoamine oxidase-B (MAO-B). Promising MAO inhibitors were further checked for the inhibition of acetylcholinesterase and neuroinflammation. Among them, compounds **3aa** and **3bc** were identified as potential multifunctional molecules eliciting submicromolar selective MAO-B inhibition, low-micromolar AChE inhibition, and the inhibition of microglial PGE_2_ production. An evaluation of their effects on memory and cognitive impairments using a passive avoidance test confirmed the in vivo activity of compound **3bc**, which showed comparable activity to donepezil. In silico molecular docking provided insights into the MAO and acetylcholinesterase inhibitory activities of compounds **3aa** and **3bc**. These findings suggest compound **3bc** as a potential lead for the further development of agents against neurodegenerative diseases.

## 1. Introduction

Neurodegenerative diseases, including Alzheimer’s disease (AD) and Parkinson’s disease (PD), impose a high global disease burden [1]. Together, they constituted the leading cause of the highest disability-adjusted life years (DALYs) and the second-highest cause of death in 2016 [2]. The fact that the currently implemented therapeutic agents are far from achieving comprehensive remedy and, moreover, are ineffective in some patients raises an urgent need for the development of novel therapeutic agents.

Neurodegenerative diseases share several commonalities, including neuronal death and degeneration that triggers abnormal levels of chemical neurotransmitters, including monoamines and acetylcholine. It is well-established that both types A and B of monoamine oxidases (MAOs), which are responsible for the metabolic oxidation of monoamine neurotransmitters, have crucial roles in the induction of neurodegeneration through the production of reactive oxygen species (ROS) [3,4,5,6]. The pathogenesis of Alzheimer’s disease (AD) is implication by MAOs and associated with the neurodegeneration of the cholinergic system. Accordingly, both monoamine oxidase inhibitors (MAOIs) and acetylcholinesterase inhibitors (AChEIs) are candidates for developing anti-Alzheimer’s-disease agents [7,8,9,10]. Meanwhile, Parkinson’s disease (PD) is mainly associated with the degeneration of dopaminergic neurons, resulting in low dopamine levels. Hence, monoamine oxidase-B (MAO-B) inhibitors not only elicit neuroprotective effects through inhibition of ROS production but also enhance dopamine levels and, thus, are a proven clinical approach for the management of PD [11]. In addition to providing protection against oxidative stress, MAOIs increase the gene expression of antiapoptotic and pro-survival proteins [3,12]. Furthermore, MAO activity is implicated in the formation of β-amyloid plaques in AD, and MAO-B is upregulated in AD [13,14]. On the other hand, the use of AChEIs is beneficial for the management of AD, and they are used off-label to alleviate cognitive symptoms in PD [15]. The combined axial roles for MAOs and AChE in the theory of multiple mechanisms of neurodegeneration stimulate the development of bifunctional molecules, inhibiting both MAO and AChE activities to combat neurodegenerative diseases.

Neuroinflammation is another component in neurodegenerative diseases such as AD and PD [16]. Microglia are the CNS resident form of macrophages, which play axial roles in neuroinflammation and are responsible for the production of inflammatory mediators such as nitric oxide (NO), PGE_2_, and cytokines. Anomalous microglia activity has been detected in neurodegenerative diseases [16]. Growing evidence has proven that alleviating neuroinflammation is a valid strategy in the treatment course of neurodegenerative diseases [17,18,19]. Interestingly, MAO-B inhibitors were reported to repress microglia-contributed neuroinflammation [20,21]. Noteworthily, the literature unveiled anti-neuroinflammatory effects for acetylcholine (ACh) mediated by microglia; hence, AChEIs might suppress neuroinflammation [22,23,24].

Considering the complex multifactorial nature of neurodegenerative diseases, multifunctional molecules modulating multiple components of these diseases could be a more effective approach [25,26,27,28]. In fact, the development of MAO inhibitors possessing multiple activities for neurodegenerative diseases has been called for [29]. Therefore, this work aimed to identify new candidate molecules inhibiting MAOs and AChE as well as microglia-mediated neuroinflammation for the development of potential agents for the management of neurodegenerative diseases.

## 2. Materials and Methods

### 2.1. Chemical Synthesis

Compounds **2a**–**2j**, **3aa**–**3ak**, and **3am**–**3bd** were prepared as reported earlier (Supplementary Information) [30,31]. Compound **3al** was prepared analogously (Supplementary Information). 

### 2.2. Monoamine Oxidases Inhibition Assay

Evaluation was conducted following standard literature protocols, as described in Supplementary Materials [32,33].

### 2.3. Acetylcholinesterase Inhibition Assay

Evaluation was conducted following standard literature protocols, as described in Supplementary Materials [34,35]. 

### 2.4. Cellular Viability and Inhibition of PGE_2_ Production Assays

Evaluations were conducted following standard literature protocols, as described in Supplementary Materials [33].

### 2.5. In Vivo Evaluations 

Evaluation was conducted following standard literature protocols, as described in Supplementary Materials [36]. Animal experimental protocols were approved by the Institutional Animal Care and Use Committee of Kyung Hee University (KHSASP-22–022, 28 March 2022).

### 2.6. Statistical Analysis

Results were analyzed by one-way analysis of variance (ANOVA) with the Newman–Keuls multiple comparisons test. Differences between groups were considered significant at *p* < 0.05. All statistical analyses were performed with Prism 7.0 software (GraphPad, La Jolla, CA, USA).

### 2.7. Molecular Modeling Studies

Studies were conducted following standard literature protocols [33,37] employing X-ray cocrystals of MAO-B with safinamide (PDB: 2V5Z); (b) MAO-A with harmine (PDB: 2Z5X); and AChE with galantamine (PDB: 4EY6). 

## 3. Results and Discussion

### 3.1. Design of Focused O^6^-Aminoalkyl Derivatives of Hispidol Analogs Library

**Figure 1:**
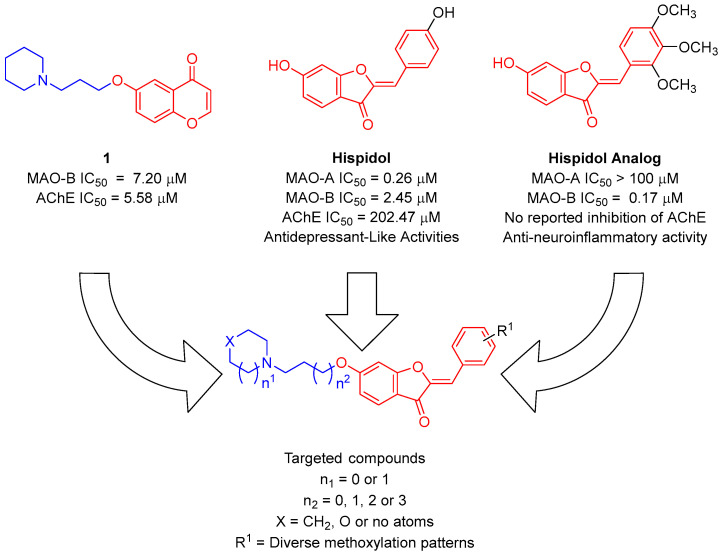
Design of *O*^6^-aminoalkyl-hispidol analogs’ focused library considering known monoamine oxidases inhibition and selectivity of hispidol as well as other methoxylated-aurones.

A growing body of literature reports supports the correlation between the high success in drug discovery and development with the implementation of natural-product-based drug discovery strategies [38,39,40,41]. Encouraged by these reports coupled with the need for new multifunctional molecules, this report aimed to identify promising compounds modulating the monoaminergic, cholinergic, and neuroinflammatory pathways implicated in neurodegenerative diseases. Recently, hispidol (Figure 1), a phytoalexin natural flavonoid isolated from legume plants including soybean, *Retama raetam***,** and *Medicago truncatula* [42,43,44], has been identified as a potential reversible MAO inhibitor with low micromolar activity and selectivity towards the inhibition of MAO-A over MAO-B. Despite the fact that some flavonoids might show AChE inhibitory activity [45], a preliminary evaluation of the AChE inhibitory activity of hispidol returned a disappointing IC_50_ value of 202.47 µM. However, an exploration of the MAO-B inhibitory activity of ring-B hydroxylated/methoxylated hispidol analogs unveiled several ring-B methoxylated analogs as highly selective potential MAO-B inhibitors with promising anti-neuroinflammatory effects [33]. Meanwhile, attaching an aminoalkyl moiety to the known chromone-based reversible MAO inhibitor was recently reported to afford compound **1** as a bifunctional MAO-B/AChE inhibitor, albeit showing micromolar activity against both targets (Figure 1) [46]. Aiming to realize a multifunctional molecule inhibiting all monoamine oxidase, acetylcholinesterase, and neuroinflammation pathways, we planned to incorporate hispidol analogs as surrogates to the chromone moiety to prepare and evaluate the activity of a focused library of *O*^6^-aminoalkyl-hispidol analogs against these pathways involved in neurodegenerative diseases. 

Recently, some *O*^6^-aminoalkyl-aurone derivatives were investigated for AD [30]. However, the aim of the previous report was limited to achieving AChEI and did not include achieving potential polypharmacological candidates. Other related *O*^6^-aminoalkyl-aurone derivatives appeared in two recent reports that explored very limited methoxylation patterns of aurone’s ring-B to provide enough SAR information and, in addition, were investigated only for either AChEI [47] or the inhibition of MAO/β-amyloid aggregation [48]. Furthermore, their MAO inhibition activity was not sufficient. Moreover, the crucial neuroinflammatory component was overlooked. Following the accredited strategy of repurposing and repositioning in drug design and discovery [49,50,51,52], a retrospective review of the previous compounds according to the design approach presented herein might suggest repurposing them to be explored for their effect on monoamine oxidases, acetylcholinesterase, and neuroinflammation, which are involved in neurodegenerative diseases.

To conduct an efficient exploration of SAR, members of the designed focused library, as shown in Figure 1, featured several structural modifications. They planned to possess diverse ring-B methoxylation patterns. Thus, molecules possessing 2′-/3′- or 4′-methoxy substituents were represented in library’s compounds to explore the outcome of a positional shift of monomethoxy substituents. In addition, different patterns of ring-B dimethoxylation were planned to be represented among library members. The designed library also encompassed members possessing a ring-B trimethoxylation pattern. Regarding the aminoalkyl moiety linked to *O*^6^-position of the incorporated hispidol analogs, and as shown in Figure 1, the amine moiety varied between diethylamine and cycloalkylamines. The explored cycloalkylamines included the five-membered pyrrolidine as well as the six-membered piperidine and morpholine. Finally, the length of the alkyl linker was varied between two, three, four, and five carbons to assess its impact on the activity.

### 3.2. Synthesis of Targeted Library Members

**Scheme 1:**
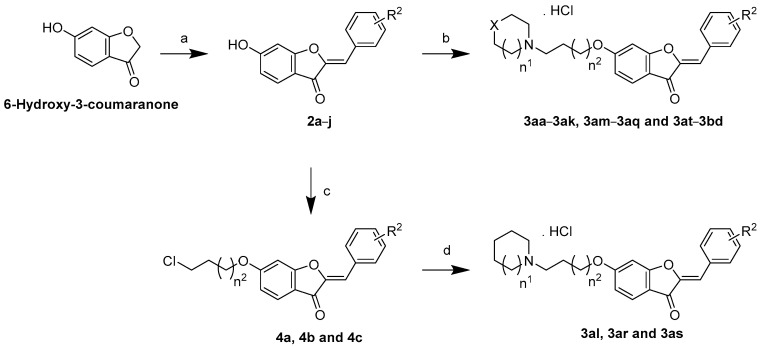
Reagents and reaction conditions: (a) 12N HCl, ethanol, 60–70 °C; (b) Appropriate aminoalkyl chloride, K_2_CO_3_, acetone, reflux; Acetyl chloride, ethanol; (c) For **5a** and **5b**: 1-bromo-4-chlorobutane, K_2_CO_3_, DMF, rt, reflux; For **5c**: 1-bromo-5-chloropentane, K_2_CO_3_, DMF, rt; (d) For **3al**: pyrrolidine, DMF, KI, reflux; For **3ar** and **3as**: piperidine, DMF, KI, reflux; Acetyl chloride, ethanol.

Concise synthesis is an important goal to achieve practical and economic synthesis [53,54,55]. In this regard, synthesis of the targeted library members was achieved in two or three sequential synthetic steps in analogy to the reported protocol as outlined in Scheme 1 [30]. First, 10 hispidol analogs **3a–j** bearing diverse methoxylation patterns were prepared via the acid-catalyzed cross-aldol condensation of the commercially available 6-hydroxy-3-coumaranone with diversely-substituted aromatic aldehydes. The targeted compounds **3aa–ak**, **3am–aq**, and **3at–bd** were accessed through the *O*^6^-alkylation of hispidol analogs **3a–j** with the appropriate commercially available aminoalkyl chloride using anhydrous potassium carbonate in refluxing acetone as a solvent. The obtained *O*^6^-aminoalkyl hispidol analogs were converted into salt form via adding acetyl chloride to ethanolic solutions, which yielded the targeted compounds as HCl salts. Regarding aminoalkyl derivatives **3al**, **3ar**, and **3as** having four or five carbon chain linkers, the phenolic hydroxyl group was first alkylated using the appropriate terminally halogenated chloroalkyl bromide employing anhydrous potassium carbonate in refluxing DMF to afford alkyl chloride derivatives **5a–c**, followed by reaction with the appropriate cyclic amine in refluxing DMF in the presence of potassium iodide. The treatment of ethanolic solutions of the products with acetyl chloride afforded the desired compounds **3al**, **3ar**, and **3as** in the form of HCl salts.

### 3.3. InVitro Biological Evaluations

#### 3.3.1. Evaluation of Inhibition of Different Monoamine Oxidase Isoforms

Two isoforms of MAOs, namely MAO-A and MAO-B, are expressed in CNS, showing different substrate specificity. Consequently, different pharmacological effects can be elicited depending on the inhibited MAO isoform. Therefore, the prepared *O*^6^-aminoalkyl-hispidol analogs were evaluated against both recombinant human MAO-A and MAO-B isoforms following the well-established spectrophotometric method [32]. The results are displayed in Table 1 and discussed in the following sections.

##### Evaluation of MAO-A Inhibitory Activity

As the members of the prepared library are *O*^6^-aminoalkyl-hispidol analogs, while hispidol was reported with sub-micromolar IC_50_ value against MAO-A, evaluations of the compounds’ inhibitory activities against recombinant human MAO-A were conducted at a 1 µM concentration to detect potential MAO-A inhibitors. Regardless of the ring-B methoxylation pattern or the *O*^6^-aminoalkyl moiety, all tested compounds showed no to very weak MAO-A inhibition that did not exceed 12.73% at the tested 1 µM concentration. Such an outcome, which contrasts hispidol’s tendency to show a potential MAO-A inhibition is, in fact, consistent with the recently found low activity of hispidol analogs as MAO-A inhibitors [33].

##### Structure-Activity Relationship of MAO-B Inhibitory Activity

The unveiled low activity against MAO-A could be beneficial in achieving selective MAO-B inhibition. As hispidol was reported to have a low micromolar IC_50_ value for the inhibition of MAO-B, equivalent to 2.45 µM, the prepared compounds were evaluated for the inhibition of activity of recombinant human MAO-B isoforms at a single dose of 10 µM. According to the results, the activity was influenced by the ring-B methoxylation pattern, the introduced amino moiety, and the length of the carbon chain linker. As shown in Table 1, among three compounds having 2′-methoxy substituents (**3aa**–**3ac**), *O*^6^-diethylaminoethyl derivative **3aa** triggered the excellent inhibition of MAO-B by 91.49%. Meanwhile, replacing the diethylamino with the cyclic pyrrolidino moiety afforded derivative **3ab** with decreased activity, which was almost half that of derivative **3aa**. Increasing the linker’s length by one carbon resulted in a further activity reduction (compounds **3ac**). Shifting the methoxy from the 2′-position in compound **3aa** to the 3′-position resulted in derivative **3ad** having almost half activity. Again, increasing the linker’s length by one carbon resulted in a further decrease of the activity (compounds **3ae**), while replacing the diethylamino with the cyclic pyrrolidino moiety to afford compound **3af** slightly decreased the activity, and when coupled with an increase in linker length by one carbon to afford compound **3ae**, it resulted in a marked activity decrement. Replacing the pyrrolidine of compound **3ag** with piperidine while retaining the same linker’s length maintained almost the same low MAO-B inhibition level (compound **3ah**), while replacing the pyrrolidine of compound **3af** with morpholine while maintaining the same linker’s length resulted in decreased activity (compound **3ai**). In contrast to the observed activity reduction in the cases of the presence of a methoxy group at the 2′- or 3′-positions, switching to the ring 4′-methoxylation pattern and incorporating a pyrrolidine moiety was associated with a slight increase in activity upon increasing the length of the alkyl chain linker (Table 1, compounds **3aj**, **3ak**, and **3al**). Meanwhile, replacing the pyrrolidine with a piperidine moiety resulted in compound **3am** eliciting nearly half the measured activity for compound **3aj**, which shares the same alkyl chain length. It was noted that compounds **3af** and **3aj** share the same pyrrolidine moiety and length of the alkyl chain but have ring-B possessed a 3′-methoxy or 4′-methoxy, respectively, and elicited almost the same activity level. Therefore, the combined ring-B methoxylation pattern, namely 3′,4′-dimethoxy, was explored maintaining the same pyrrolidine moiety and length of the linker (compound **3an**) or replacing pyrrolidine with piperidine but retaining the same linker’s alkyl chain length (compound **3ap**). As revealed from the results, both compounds **3an** and **3ap** possessed almost the same activity level, which was slightly higher relative to compounds **3af** and **3aj**. Despite the decrease in activity upon increasing the linker’s alkyl chain length, it was marginal in the case of pyrrolidine derivative **3ao** compared with compound **3an**. The activity reduction upon increasing the linker’s alkyl chain length was highly remarkable in the case of piperidine derivatives **3aq**, **3ar**, and **3as**, which showed very low activities relative to compound **3ap**. Changing the 3′,4′-dimethoxylation pattern of the ring-B of compound **3an** into 3′,5′-dimethoxylation pattern to afford compound **3at** maintaining the same pyrrolidine moiety and the two-carbon chain linker slightly decreased the activity, and when coupled with increasing the linker’s length to a three-carbon chain, it resulted in further decrease of the activity (compound **3au**). An exploration of other ring-B dimethoxylation patterns while retaining two-carbon chain linker and pyrrolidine moiety showed that, among them, the 2′,5′-dimethoxy derivative **3ax** was the most active, while the 2′,4′-dimethoxy derivative **3aw** was less active, and the 2′,3′-dimethoxy derivative **3av** was almost inactive. Again, increasing the linker’s alkyl chain afforded, in general, compounds with lower activities (Table 1, compounds **3ay**, **3az**, and **3ba**). Finally, an exploration of ring-B trimethoxylation patterns revealed excellent activity for the 3′,4′,5′-trimethoxlated compound **3bc** but low activity for the 2′,3′,4′-trimethoxlated compound **3bb**; both of them have pyrrolidine moieties and two-carbon alkyl chain linkers. Modifying the pyrrolidine moiety of the excellently active compound **3bc** into piperidine moiety afforded the poorly active compound **3bd**. Collectively, it might be inferred that a short alkyl chain linker results in better activity, possibly because of substrate pocket size limitations. In addition, the combined ring-B methoxylation pattern and the type of amino moiety together dictate the elicited MAO-B inhibitory activity. Among the explored patterns, a combination of diethylamino and 2′-methoxy substitution patterns as well as pyrrolidine and 3′,4′,5′-trimethoxy substitution patterns coupled with two-carbon chain linkers afforded compounds showing an excellent activity.

##### Evaluation of IC_50_ and MAO-B Selectivity

Potency and selectivity are two important criteria that should be assessed for promising compounds. The most active compounds, which triggered almost ≥50 inhibition of MAO-B activity, were evaluated for potency by determining the MAO-B IC_50_ values employing five doses. In addition, MAO-A inhibition at the high concentration of 100 µM was determined as well as MAO-A IC_50_ values to calculate MAO-B inhibition selectivity indices. As shown in Table 2, compounds **3al** and **3ap** inhibited MAO-A by almost 15% and 23% at the high 100 µM concentrations and showed high IC_50_ values, indicating low potencies and selectivity indices for compounds **3al** and **3ap**. The calculated selectivity indices were >12 and >9.8 for compounds **3al** and **3ap**, respectively. Despite compound **3ax** showing an approximately 28% inhibition of MAO-A at the high concentration of 100 µM, it was a more potent MAO-B inhibitor compared with compounds **3al** and **3ap**, which showed an IC_50_ of 3.16 µM, resulting in a better MAO-B selectivity index of >32. However, the potency of compound **3ax** was in low micromolar range, which might require further optimization. Fortunately, compounds **3bc** and **3aa** elicited low IC_50_ values in the submicromolar range, reflecting good potencies. Thus, compound **3bc** showed IC_50_ of 0.34 µM, which is almost three times the measured IC_50_ value of safinamide, the reference standard drug. The calculated >289 selectivity index for MAO-B inhibition by compound **3bc** was excellent. Meanwhile, compound **3aa** was also a good selective MAO-B inhibitor, eliciting IC_50_ for MAO-B inhibition in the submicromolar range by 0.96 µM, which was around 8.6 times the measured IC_50_ value of safinamide and possessed a good selectivity index of >104.

#### 3.3.2. Evaluation of Acetylcholinesterase Inhibitory Activity 

While AChE inhibition was assessed previously for some members of these compounds, the results were unknown for other compounds. Accordingly, AChE inhibition was assessed for compounds that emerged as promising MAO-B inhibitors whenever AChE inhibition was unreported, adopting the same reported assay protocol and employing galantamine as a reference standard AChEI [30]. As in Table 2, all promising compounds were more potent than galantamine as AchEIs. Thus, compound **3bc**, which was the most potent and selective MAO-B inhibitor, elicited a potential AChE inhibition with a measured low IC_50_ value of 1.56 µM. Hence, compound **3bc** possesses almost three times the potency of the reference standard galantamine. In addition, compound **3aa**, which was the second-most potent and selective MAO-B inhibitor, also triggered a potential AChE inhibition. It showed a measured IC_50_ of 2.67 µM, which is nearly 1.8 times the potency of the reference galantamine. Despite the lower MAO-B potency and selectivity of compounds **3ax** and **3al** as MAO-B inhibitors, they possessed potential AChEI, showing equipotent activities to compound **3bc**. Finally, compound **3ap** was the most potent AChEI among these five compounds, but it was the least potent and selective as a MAO-B inhibitor. As the ligand binding pockets of AChE and MAO-B are different, the absence of a correlation between AChE and MAO-B inhibitions is a logical outcome. However, it is possible to develop potential ligands interacting with both different enzymes, as found for compounds **3aa** and **3bc**.

#### 3.3.3. Evaluation of Anti-Neuroinflammatory Activity 

**Figure 2:**
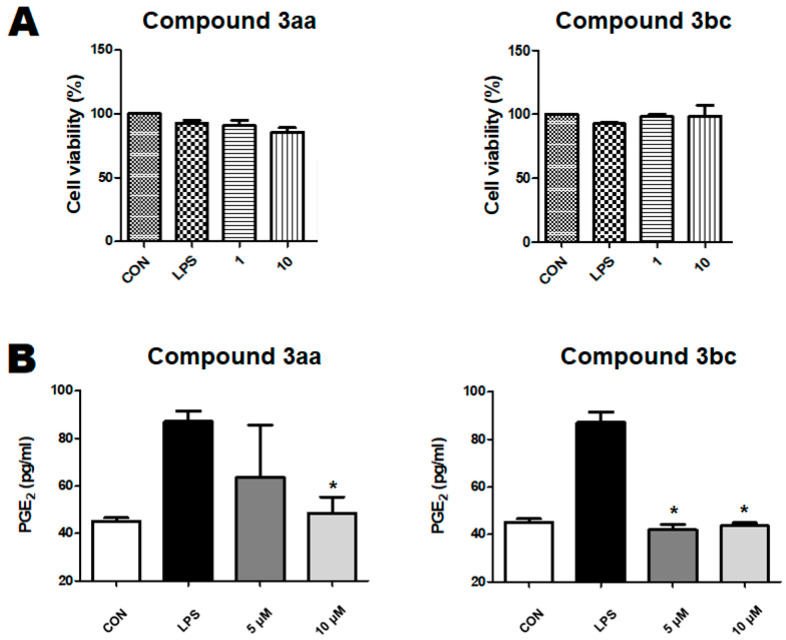
Dose-dependent effect of compounds **3aa** and **3bc** on microglial BV2 cells: (**A**) Viability assessment (mean ± SD) of BV2 cells in presence of LPS and variable doses of compounds **3aa** and **3bc**; (**B**) Dose-dependent inhibition (mean ± SD) of LPS-induced BV2 cell production of PGE_2_ variable doses of compounds **3aa** and **3bc**. Results were analyzed by ANOVA. * *p* < 0.05 versus LPS-treated control group.

To advance the compounds for further cellular evaluations, a preliminary check for the absence of undesirable cytotoxic effects might be needed. This might enable the early elimination of toxic compounds as well as the reduction of non-specific responses arising from cell death. Accordingly, the impact of compounds **3aa** and **3bc** on the cellular viability of microglial BV2 cells was evaluated. The results indicated the absence of cytotoxic activity in both compounds **3aa** and **3bc** on a cellular viability of up to 10 micromolar concentrations (Figure 2A). Consequently, compounds **3aa** and **3bc** were advanced for the evaluation of their impact on BV2 cells’ induced production of PGE_2_, a known mediator of neuroinflammation linked to neurodegenerative diseases. As shown in Figure 2B, LPS treatment induced an increase in PGE_2_ production, suggesting the initiation of a neuroinflammatory reaction. Such increased PGE_2_ production was alleviated and returned to almost normal levels after 10 micromolar doses of compounds **3aa** or **3bc**. Meanwhile, the normal PGE_2_ production level was still by the lower five micromolar concentrations of compound **3bc,** while the attrition of the inhibitory effect was observed in the case of compound **3aa**. Together, these findings demonstrate that both compounds **3aa** and **3bc** are multifunctional compounds showing potential anti-neuroinflammatory effects at 10 micromolar doses in addition to their MAO-B and AChE inhibitory effects.

### 3.4. In Vivo Evaluation of Cognitive Deficit Amelioration

**Figure 3:**
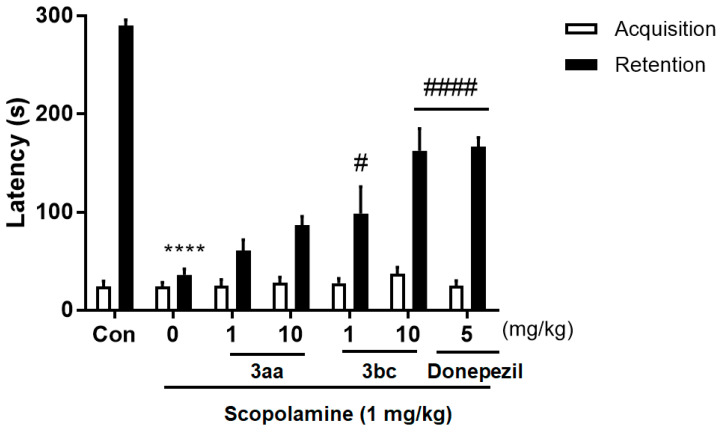
In vivo Evaluation of effects (mean ± SD) of compounds **3aa** and **3bc** (1 and 10 mg/kg, p.o.) on scopolamine-induced cognitive deficits (1 mg/kg, i.p.) in comparison with donepezil (5 mg/kg, p.o.). Results were analyzed by ANOVA. *n* = 10 per group, **** *p* < 0.0001 versus vehicle control group, # *p* < 0.05 and #### *p* < 0.0001 versus scopolamine treated group.

To evaluate the in vivo effects of compounds **3aa** and **3bc** on cognitive deficits, an in vivo model of scopolamine-induced cognitive deficits in rodents was employed using a passive avoidance test. The passive avoidance task is a fear-motivated test intended to assess the effects of deteriorating cognitive functions caused by neurodegenerative diseases on the capacity to learn and memorize [56]. First, mice underwent acquisition to learn avoidance. Next, the capacity of mice to memorize and retain the learned avoidance was assessed. As shown in Figure 3, the administration of 1 and 10 mg/kg doses of compound **3bc** significantly enhanced latency in the retention experiment. These results revealed that the 10 mg/kg dose of compound **3bc** was as effective as donepezil in ameliorating cognitive deficits. Meanwhile, compound **3aa** did not show significant amelioration of cognitive deficits. These findings suggest that compound **3bc** could potentially alleviate the impairment of memory and cognitive functions because of neurodegenerative diseases.

### 3.5. In Vivo Evaluation of Antidepressant Activity

**Figure 4:**
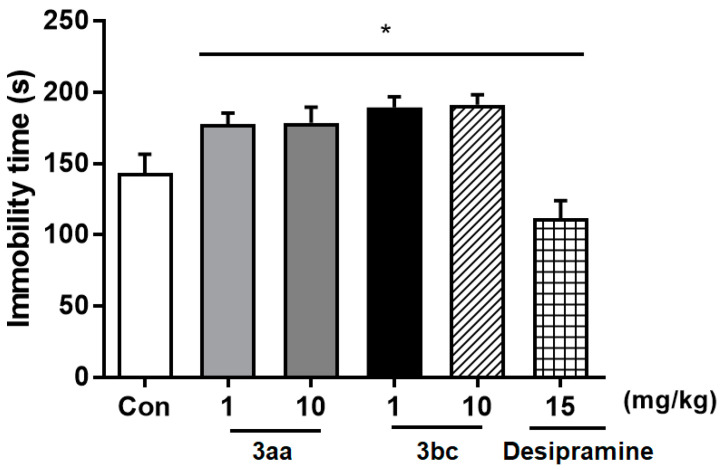
In vivo evaluation of effects (mean ± SD) of compounds **3aa** and **3bc** (1 and 10 mg/kg, p.o.) on immobility time in comparison with 15 mg/kg desipramine (15 mg/kg, i.p.). Results were analyzed by ANOVA. n = 10 per group, * *p* < 0.05 versus control group.

Recently, ladostigil, an investigational AChEI/selective MAO-B inhibitor, has been found to elicit antidepressant-like effects and was proposed for treatment of depression and psychiatric disorders associated with neurodegenerative diseases [57]. This suggested the evaluation of the antidepressant-like activity of compounds **3aa** and **3bc** using an in vivo model of forced-swim test in rodents: a well-recognized model for evaluation of antidepressants [58]. Thus, an acute-treatment forced-swim test was applied to six rat groups: an untreated control group, four groups treated with 1 and 10 mg/Kg oral doses of compounds **3aa** and **3bc**, respectively, and a group treated with 15 mg/kg intraperitoneal dose of the antidepressant drug desipramine. As shown in Figure 4, the results showed that compounds **3aa** and **3bc** did not show antidepressant-like effects in the acute-treatment model. Despite this seems to be in line with the reported results of ladostigil which indicated that it shows antidepressant-like effects in chronic-treatment model but not in acute-treatment model, compounds **3aa** and **3bc** significantly increased immobility time in the conducted acute-treatment model. Therefore, they were not advanced for evaluation in the chronic-treatment model. Although these findings suggest that compounds **3aa** and **3bc** might have no therapeutic effects on depression and psychiatric disorders associated with neurodegenerative diseases, compound **3bc** might have potential activity against the neurodegenerative diseases themselves. However, the found increase in immobility time suggests that the use of this class of compound be cautioned in neurodegenerative diseases that are comorbid with depression. 

### 3.6. In Silico Simulation Study

**Figure 5:**
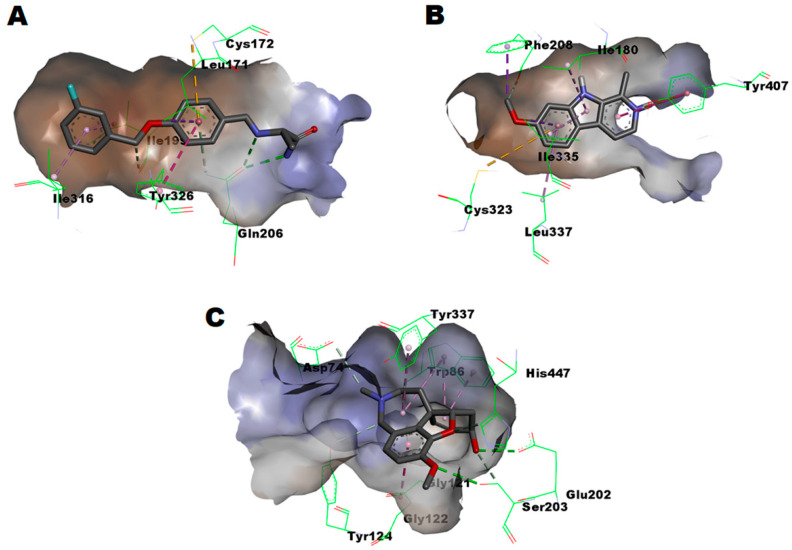
(**A**) Interactions network of safinamide within MAO-B pocket as found in the co-crystalized complex (PDB: 2V5Z); (**B**) Interactions network of harmine within MAO-A pocket as found in the co-crystalized complex (PDB: 2Z5X); (**C**) Interactions network of galantamine within AChE substrate pocket as found in the co-crystalized complex (PDB: 4EY6).

To obtain insights into the molecular interactions of the potential compounds **3aa** and **3bc**, in silico calculations were addressed to anticipate their possible binding modes to MAO-B (PDB: 2V5Z), MAO-A (PDB: 2Z5X), and AChE (PDB: 4EY6). Structural studies indicated that MAO-A has a 550 Å^3^ substrate-pocket, which is relatively smaller than the elongated 700 Å^3^ substrate-pocket of MAO-B, which is characterized by the presence of gating amino acid residues, namely Ile199 and Tyr326, that can close to form a bipartite cavity or open to accommodate linear ligands such as safinamide (Figure 5A, PDB code: 2V5Z). In the case of MAO-A, residues Ile335 and Phe208 serve as gating amino acids in a similar fashion to Tyr326 and Ile199 of MAO-B. In addition, both MAOs have functionally important two-cage aromatic amino acid residues in front of flavin (Tyr398 and Tyr435 for MAO-B and Tyr407 and Tyr444 for MAO-B). Depending on size and shape differences as well as the involvement of gating residues, some substrates, such as harmine (Figure 5B, PDB code: 2Z5X), are selective MAO-A inhibitors, while others are selective MAO-B inhibitors [59,60,61]. On the other hand, characterization studies showed that AChE has much larger binding site in which there is an important catalytic triad of Ser203, His447, and Glu334 residues, which is crucial for the functional activity of AChE in conjugation with an oxyanion hole formed of the backbone NH groups of Gly121, Gly122, and Ala204 [62]. As illustrated in Figure 5C, the cocrystals of AChE-galantamine (PDB: 4EY6) revealed that the galantamine ether bridge interacts with His447 in addition to the methoxy function interaction with Ser203; both residues belong to the catalytic triad. Furthermore, amide–π stacking was found between galantamine and the peptide bonds of Gly121–Gly122 in the oxyanion hole. Moreover, other hydrophobic and hydrogen bonding interactions were found for other residues with the galantamine’s skeleton and functional groups. 

**Figure 6:**
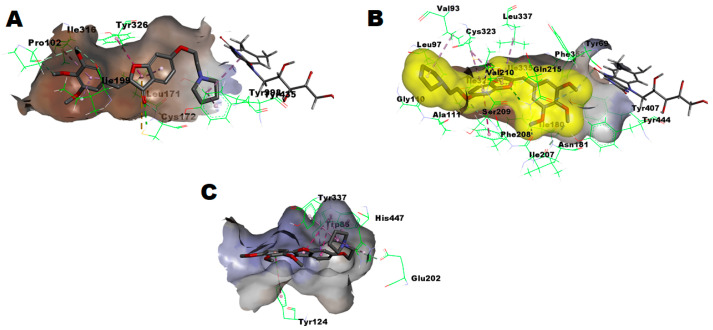
(**A**) Predicted binding mode of compound **3bc** within MAO-B pocket (PDB: 2V5Z); (**B**) Predicted binding mode of compound **3bc** within MAO-A pocket (PDB: 2Z5X): Yellow surface represents the solvent accessible surface of compounds **3bc**; (**C**) Predicted binding mode of compound **3bc** within AChE pocket (PDB: 4EY6).

As illustrated in Figure 6A, the potent selective MAO-B inhibitor, compound **3bc**, perfectly fitted within the MAO-B substrate pocket, showing an estimated good energy score of −8.56548 Kcal/mol and establishing an intricate network of favorable interactions. The predicted binding mode showed that the pyrrolidine moiety is sandwiched between the two aromatic cage amino acids, namely Tyr398 and Tyr435, establishing one π–alkyl hydrophobic interaction per aromatic cage residue and a third π–π hydrophobic interaction with the FAD moiety. In addition, the gating Tyr326 amino acid residue interacted favorably with ring-C of the aurone part, while the other gating Ile199 amino acid residue showed a favorable interaction with the 3′-methoxy substituent at ring-B. Furthermore, several other residues established favorable interactions, which, in conjugation with the aforementioned key interactions, provide evidence of the excellent inhibition of MAO-B. On the contrary, compound **3bc** poorly fitted within the MAO-A substrate pocket, clashing due to the pocket size limitation (Figure 6B) and showing a low energy score of only −5.5883 Kcal/mol. Furthermore, no favorable interactions with the aromatic cage amino acids of MAO-A, namely Tyr407 and Tyr444, were observed in the predicted binding mode. Due to the absence of these crucial interactions, the low energy score, and the clash with the pocket boundary distant to FAD, the very limited inhibition of MAO-A activity and, consequently, the excellent MAO-B selectivity that we found is understandable. In regard to AChE, the second-best predicted pose of compound **3bc** within the AChE pocket showed an energy score of −8.89504 Kcal/mol and established favorable interactions via the pyrrolidine moiety with the crucial catalytic triad member His447, as well as with other residues (Figure 6C). In addition, the ethyl linker interacted favorably with Glu202 next to both the oxyanion hole residue Ala204 and the catalytic triad member Ser203. In lieu of the established interactions, the good AChE inhibitory activity of compound **3bc** is understandable.

**Figure 7:**
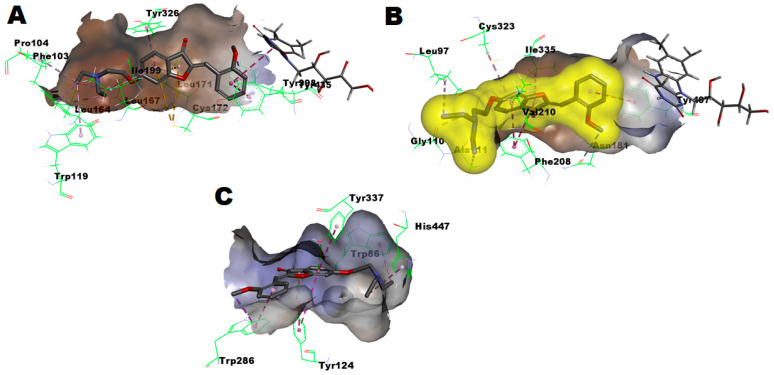
(**A**) Predicted binding mode of compound **3aa** within MAO-B pocket (PDB: 2V5Z) (**B**) Predicted binding mode of compound **3aa** within MAO-A pocket (PDB: 2Z5X): Yellow surface represents the solvent accessible surface of compounds **3aa**; (**C**) Predicted binding mode of compound **3aa** within AChE pocket (PDB: 4EY6).

As illustrated in Figure 7A, in silico calculations predicted that the potent selective MAO-B inhibitor compound **3aa** perfectly fits within the substrate pocket of MAO-B but in a flipped mode relative to compound **3bc,** showing good energy score of -9.41281 Kcal/mol. Thus, ring-B of the aurone part was sandwiched between two aromatic cage amino acids, Tyr398 and Tyr435, establishing one π–π hydrophobic interaction per aromatic cage residue, a third π–πl hydrophobic interaction with the FAD moiety, and a fourth π–σ hydrophobic interaction between the 2′-methoxy substituent at ring-B and aromatic cage Tyr398 residue. Meanwhile, the diethylamino moiety was directed towards the distant side to the aromatic cage and showed a favorable interaction with the gating residue Ile199 as well as other residues. Moreover, ring-A of the aurone moiety also interacted favorably with the gating residue Ile199 as well as the other gating residue Tyr326. According to these calculated interactions with the crucial features of MAO-B and the overall good energy score, the potential activity of compound **3aa** against MAO-B is reasonable. In contrast to MAO-B, a poor-fitting, low energy score of −5.1401 Kcal/mol and poor interactions were predicted for compound **3aa** within the substrate pocket of MAO-A. As illustrated in Figure 7B, the best predicted pose showed clashing with the substrate-pocket at the distant boundary to the aromatic cage. In addition, only one far interaction >5.7 Å distance was established with one aromatic cage Tyr407 residue. Due to the lack of crucial interactions, the calculated low energy score coupled, and the clash with the pocket’s boundary distant to FAD and the aromatic cage, the measured limited inhibition of MAO-A activity and, consequently, the excellent MAO-B selectivity is a logical outcome. Similar to compound **3bc**, the results of the in silico docking of compound **3aa** to AChE showed that the second-best predicted pose showed a favorable energy score of −7.80987 Kcal/mol, which is less than that of compound **3bc,** which is in agreement with the difference in potency of AChE inhibitory activity of the two compounds. Analogous to compound **3bc**, compound **3aa** established favorable interactions via the diethylamino moiety with the crucial catalytic triad member His447, as well as with other residues (Figure 7C). Accordingly, the good AChE inhibitory activity of compound **3aa** is comprehensible.

## 4. Conclusions

To attain a multifunctional molecule inhibiting the commonalities of neurodegenerative disorders, including ROS, the impaired neurochemical transmission of monoamines, and acetylcholine esterase and neuroinflammation, a series of *O*^6^-aminoalkyl derivatives of analogs of the natural product hispidol were prepared and evaluated for MAO and AChE inhibition. Several compounds showed potential inhibition of MAO and AChE. Among these, compounds **3aa** and **3bc** triggered selective MAO-B inhibition with decent selectivity indices over 104 and 289, respectively. An evaluation of the anti-neuroinflammatory activity of the promising compounds **3aa** and **3bc** demonstrated their potential capability to inhibit induced microglial production of PGE_2_ without impacting cellular viability. The developed multifunctional compounds **3aa** and **3bc** were advanced for an in vivo evaluation of their effects on impairment of memory and cognitive functions and antidepressant-like activity in a forced swim test. The results indicated that the more potent and selective compound **3bc** can improve memory and cognitive function impairment, while the less potent and less selective compound **3aa** has little effect. Neither **3aa** nor **3bc** showed antidepressant-like activity in the acute-treatment model of the forced swim test, but the increase in immobility time could be a sign that this class of compound might be cautioned in neurodegenerative diseases that are comorbid with depression, possibly because of AChEIs’ contraindication for mood disorders. Finally, an in silico docking study provided insights into the MAO and AChE inhibitory activity of compounds **3aa** and **3bc**. Collectively, our efforts present compound **3bc** as a multifunctional molecule inhibiting MAO-B, AChE, and neuroinflammation for the possible management of neurodegenerative diseases.

## Figures and Tables

**Table 1:**
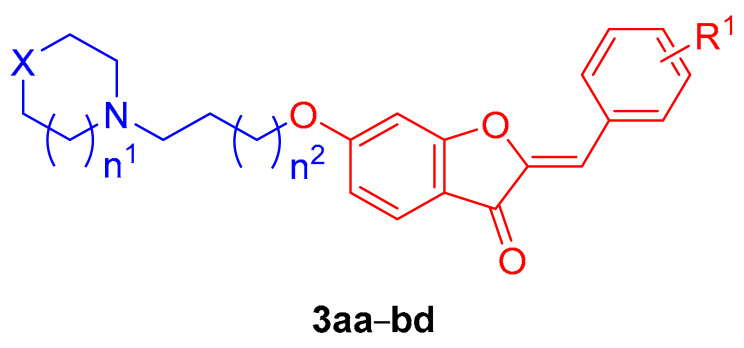
Results of MAO-A and MAO-B inhibition by the synthesized compounds (**3aa**–**3bd**).

Compound	X	n^1^	n^2^	R^1^	% Inhibition	
					MAO-A ^1^	MAO-B ^2^
3aa	No atoms	1	0	2′-methoxy	NI ^3^	91.49 ± 0.27
3ab	CH_2_	0	0	2′-methoxy	NI	43.00 ± 0.55
3ac	CH_2_	0	1	2′-methoxy	1.31 ± 0.82	32.27 ± 0.23
3ad	No atoms	1	0	3′-methoxy	NI	46.09 ± 0.11
3ae	No atoms	1	1	3′-methoxy	NI	18.76 ± 0.45
3af	CH_2_	0	0	3′-methoxy	NI	39.11 ± 0.96
3ag	CH_2_	0	1	3′-methoxy	12.24 ± 1.05	16.99 ± 0.11
3ah	CH_2_	1	1	3′-methoxy	NI	18.07 ± 1.83
3ai	O	1	0	3′-methoxy	4.1 ± 0.48	27.87 ± 0.37
3aj	CH_2_	0	0	4′-methoxy	3.26 ± 0.75	39.26 ± 0.03
3ak	CH_2_	0	1	4′-methoxy	9.83 ± 0.64	45.64 ± 0.08
3al	CH_2_	0	2	4′-methoxy	NI	49.80 ± 0.02
3am	CH_2_	1	0	4′-methoxy	0.92 ± 0.83	20.56 ± 0.79
3an	CH_2_	0	0	3′,4′-dimethoxy	NI	48.06 ± 1.46
3ao	CH_2_	0	1	3′,4′-dimethoxy	NI	39.21 ± 0.58
3ap	CH_2_	1	0	3′,4′-dimethoxy	NI	50.81 ± 0.44
3aq	CH_2_	1	1	3′,4′-dimethoxy	NI	4.51 ± 1.30
3ar	CH_2_	1	2	3′,4′-dimethoxy	NI	3.89 ± 1.63
3as	CH_2_	1	3	3′,4′-dimethoxy	NI	4.76 ± 1.91
3at	CH_2_	0	0	3′,5′-dimethoxy	NI	36.84 ± 0.44
3au	CH_2_	0	1	3′,5′-dimethoxy	NI	21.20 ± 0.30
3av	CH_2_	0	0	2′,3′-dimethoxy	12.56 ± 0.32	2.61 ± 1.78
3aw	CH_2_	0	0	2′,4′-dimethoxy	12.73 ± 0.54	41.64 ± 1.03
3ax	CH_2_	0	0	2′,5′-dimethoxy	NI	65.05 ± 0.43
3ay	CH_2_	0	1	2′,3′-dimethoxy	NI	4.37 ± 0.90
3az	CH_2_	0	1	2′,4′-dimethoxy	NI	35.87 ± 0.17
3ba	CH_2_	0	1	2′,5′-dimethoxy	NI	20.33 ± 0.49
3bb	CH_2_	0	0	2′,3′,4′-trimethoxy	NI	23.90 ± 0.81
3bc	CH_2_	0	0	3′,4′,5′-trimethoxy	NI	99.30 ± 0.38
3bd	CH_2_	1	0	3′,4′,5′-trimethoxy	NI	27.93 ± 0.36

^1^ Percent inhibition (±sem values) of enzyme activity at a single dose concentration of 1 µM., ^2^ Percent inhibition (±sem values) of enzyme activity at a single dose concentration of 10 µM., ^3^ NI: no measured inhibition.

**Table 2:**
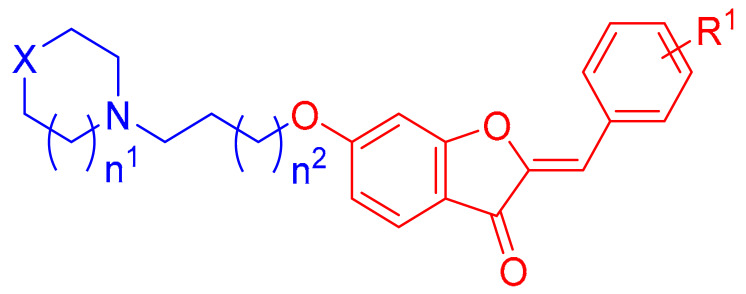
IC_50_ values for MAO-A, MAO-B, and AChE as well as MAO-B inhibition selectivity indices for the most promising compounds.

Compound	X	n^1^	n^2^	R^1^	MAO-A		MAO-B	MAO-B Selectivity Index	AChE IC_50_ (µM) ^3^
					% Inhibition ^1^	IC_50_ (µM) ^2^	IC_50_ (µM) ^2^		
3aa	No atoms	1	0	2′-methoxy	27.18 ± 2.94	>100	0.96 ± 0.004	>104	2.67 ± 0.05
3al	CH_2_	0	2	4′-methoxy	14.89 ± 3.46	>100	8.65 ± 0.196	>12	1.42 ± 0.09
3ap	CH_2_	1	0	3′,4′-dimethoxy	22.52 ± 0.04	>100	10.18 ± 0.099	>9.8	0.40 ± 0.03 [30]
3ax	CH_2_	0	0	2′,5′-dimethoxy	27.70 ± 4.95	>100	3.16 ± 0.036	>32	1.49 ± 0.31 [30]
3bc	CH_2_	0	0	3′,4′,5′-trimethoxy	21.37 ± 3.51	>100	0.34 ± 0.002	>289	1.56 ± 0.07 [30]
Safinamide					ND	ND	0.11 ± 0.004	ND	—
Clorgyline					100.49 ± 0.03	0.005 ± 0.0002	ND	ND	—
Galantamine					ND	ND	ND	ND	4.69 ± 0.12 [30]

^1^ Percent inhibition (±sem values) of MAO-A activity at a single dose concentration of 100 µM., ^2^ IC_50_: Concentration (±sem values) of the synthesized compound expressed in µM value that inhibits 50% of MAO-A/MAO-B activity., ^3^ IC_50_: Concentration (±SD values) of the synthesized compound expressed in µM value that inhibits 50% of AChE activity.

## Data Availability

Data is contained within the article or Supplementary Material.

## Supplementary Materials

The following supporting information can be downloaded at: https://www.mdpi.com/article/10.3390/antiox12051033/s1, Experimental protocols.
