# Supplementary material for: Synthesis and Biological Evaluation of O6-Aminoalkyl-Hispidol Analogs as Multifunctional Monoamine Oxidase-B Inhibitors towards Management of Neurodegenerative Diseases
Source: Antioxidants (Basel). 2023 Apr 29;12(5):1033. doi: 10.3390/antiox12051033 (PMC10215199; doi:10.3390/antiox12051033)
Supplement: Supplementary file 1 [file antioxidants-12-01033-s001.zip › antioxidants-2364835-supplementary.pdf]

Supplementary information for

Synthesis and biological evaluation of *O*[6]-aminoalkyl-hispidol analogs as multifunctional monoamine oxidase-B inhibitors towards management of neurodegenerative diseases

Ahmed H.E. Hassan [1,2], Hyeon Jeong Kim [3], Keontae Park [4], Yeonwoo Choi [5], Suyeon Moon [5], Chae Hyeon Lee [5], Yeon Ju Kim [5], Soo Bin Cho [5], Min Sung Gee [5], Danbi Lee [5], Jong-Hyun Park [3], Jong Kil Lee [5], Jong Hoon Ryu [6], Ki Duk Park [3] and Yong Sup Lee [2,5,*]

[1] *Department of Medicinal Chemistry, Faculty of Pharmacy, Mansoura University, Mansoura 35516, Egypt*

[2] *Medicinal Chemistry Laboratory, Department of Pharmacy, College of Pharmacy, Kyung Hee University, 26 Kyungheedae-ro, Seoul 02447, Republic of Korea*

[3] *Convergence Research Center for Diagnosis, Treatment and Care System of Dementia, Korea Institute of Science and Technology (KIST), Seoul 02792, Republic of Korea*

[4] *Department of Biomedical and Pharmaceutical Sciences, Kyung Hee University, 26 Kyungheedae-ro, Dongdaemun-gu, Seoul, 02447, Republic of Korea*

[5] *Department of Fundamental Pharmaceutical Science, Graduate School, Kyung Hee University, 26 Kyungheedae-ro, Dongdaemun-gu, Seoul, 02447, Republic of Korea*

[6] *Department of Oriental Pharmaceutical Science College of Pharmacy, Kyung Hee University, Seoul 02447, Republic of Korea*

[*]Corresponding author: Prof. Dr. Yong Sup Lee (email: kyslee@khu.ac.kr)

1. Chemistry

All solvents and reagents have been purchased from commercial suppliers and used without any further purification. NMR spectra were acquired on Bruker Avance 400 spectrometer (400 MHz). ^{1}H NMR spectra were referenced to tetramethylsilane (δ = 0.00 ppm) as an internal standard. High resolution mass spectra (HRMS) were recorded on Jeol AccuTOF (JMS-T100TD). TLC was carried out using glass sheets pre-coated with silica gel 60 F_{254} (Merk).

1.1.1. General method for acid-catalyzed cross-aldol condensation to prepare hispidol analogs

HCl was added dropwise to a solution of the appropriate 3-coumaranone derivative in methanol or ethanol. After complete dissolution, the appropriate benzaldehyde derivative was added dropwise. The mixture was heated at 60 °C for the specified time. After completion of the reaction (TLC), the reaction mixture was diluted with water, filtered and the collected precipitate was collected by filtration and dried under reduced vacuum before purification of the obtained crude products using column chromatography.

(*Z*)-6-Hydroxy-2-(2-methoxybenzylidene)benzofuran-3(2*H*)-one (2a) [1]

Compound **2a** was obtained from 6-hydroxy-3-coumaranone and 2-anisaldehyde. Yield 98%.

^{1}H NMR (400 MHz, DMSO-*d*6) δ 11.17 (brs, 1H), 8.15 (dd, 1H, *J* = 6.3, 1.4 Hz), 7.62 (d, 1H, *J* = 8.0 Hz), 7.43 (m, 1H), 7.12–7.08 (m, 2H), 7.06 (s, 1H), 6.79 (d, 1H, *J* = 1.8 Hz), 6.72 (dd, 1H, *J* = 8.0, 1.8 Hz), 3.89 (s, 3H); ^{13}C NMR (100 MHz, DMSO-*d*6): δ 181.3, 167.8, 166.4, 158.0, 147.3, 131.4, 130.9, 125.9, 120.8, 120.2, 133.0, 112.7, 111.4, 103.6, 98.6, 55.7.

(*Z*)-6-Hydroxy-2-(3-methoxybenzylidene)benzofuran-3(2*H*)-one (2b) [1]

Compound **2b** was obtained from 6-hydroxy-3-coumaranone and 3-anisaldehyde. Yield 61%.

^{1}H NMR (400 MHz, DMSO-*d*6) δ 11.27 (brs, 1H), 7.61 (d, 1H, *J* = 8.4 Hz), 7.52 (d,

1H, J = 8.0 Hz), 7.47 (d, 1H, J = 2.1 Hz), 7.39 (t, 1H, J = 8.0 Hz), 6.99 (dd, 1H, J = 8.0, 2.1 Hz), 6.79 (d, 1H, J = 1.8 Hz), 6.74 (s, 1H), 6.71 (dd, 1H, J = 8.4, 1.8 Hz), 3.80 (s, 3H); ^{13}C NMR (100 MHz, DMSO-d_6): δ 181.9, 168.4, 167.1, 159.8, 147.9, 133.7, 130.4, 126.4, 123.9, 116.7, 115.8, 113.6, 113.1, 110.7, 99.1, 55.6.

(*Z*)-6-Hydroxy-2-(4-methoxybenzylidene)benzofuran-3(2*H*)-one (2c) [1]

Compound **2c** was obtained from 6-hydroxy-3-coumaranone and 4-anisaldehyde. Yield 93%.

^{1}H NMR (400 MHz, DMSO-d_6) δ 11.18 (brs, 1H), 7.98 (d, 2H, J = 8.8 Hz), 7.68 (d, 1H, J = 8.4 Hz), 7.12 (d, 2H, J = 8.8 Hz), 6.86 (d, 1H, J = 1.8 Hz), 6.84 (s, 1H), 6.79 (dd, 1H, J = 8.4, 1.8 Hz), 3.89 (s, 3H); ^{13}C NMR (100 MHz, DMSO-d_6): δ 181.7, 168.1, 166.7, 160.9, 146.6, 133.4, 126.2, 125.0, 115.0, 113.5, 113.3, 111.2, 99.0, 55.7.

(*Z*)-2-(3,4-Dimethoxybenzylidene)-6-hydroxybenzofuran-3(*2H*)-one (2d) [1]

Compound **2d** was obtained from 6-hydroxy-3-coumaranone and 3,4-dimethoxybenzaldehyde. Yield 88%.

^{1}H NMR (400 MHz, DMSO-d_6) δ 11.10 (brs, 1H), 7.64–7.57 (m, 3H), 7.10 (d, 1H, J = 8.8 Hz), 6.81 (d, 1H, J = 1.8 Hz), 6.77 (s, 1H), 6.73 (dd, 1H, J = 8.4, 1.8 Hz), 3.84 (s, 6H); ^{13}C NMR (100 MHz, DMSO-d_6): δ 181.6, 168.0, 166.7, 150.8, 149.1, 146.6, 126.2, 125.5, 125.2, 114.6, 113.4, 113.0, 112.3, 111.6, 99.0, 55.9.

(*Z*)-2-(3,5-Dimethoxybenzylidene)-6-hydroxybenzofuran-3(2*H*)-one (2e) [1]

Compound **2e** was obtained from 6-hydroxy-3-coumaranone and 3,5-dimethoxybenzaldehyde. Yield 83%.

^{1}H NMR (400 MHz, Pyridine-d_5): δ 7.82 (d, J = 8.4 Hz, 1H), 7.29 (d, J = 2.1 Hz, 2H), 7.19 (s, 1H), 7.02 (dd, J = 5.2, 1.8 Hz, 1H), 6.93 (dd, J = 6.5, 1.8 Hz, 1H), 6.70 (dd, J = 2.1 Hz, 1H), 3.72 (s,

6H); ^{13}C NMR (100 MHz, Pyridine-d_5): δ 182.2, 168.9, 167.9, 161.2, 148.5, 134.6, 126.1, 133.5, 110.6, 109.3, 102.0, 99.4, 55.1.

(*Z*)-2-(2,3-Dimethoxybenzylidene)-6-hydroxybenzofuran-3(2*H*)-one (2f) [1]

Compound **2f** was obtained from 6-hydroxy-3-coumaranone and 2,3-dimethoxybenzaldehyde. Yield 97%.

^{1}H NMR (400 MHz, DMSO-d_6): δ 7.80 (dd, J = 8.0, 1.6 Hz, 1H), 7.65 (d, J = 8.4 Hz, 1H), 7.24 (t, J = 8.0 Hz, 1H), 7.17 (dd, J = 8.0, 1.6 Hz, 1H), 6.98 (s, 1H), 6.79 (d, J = 1.8 Hz, 1H), 6.74 (dd, J = 8.4, 1.8 Hz, 1H), 3.86 (s, 3H), 3.83 (s, 3H); ^{13}C NMR (125 MHz, DMSO-d_6): δ 182.0, 168.5, 167.2, 153.1, 152.7, 148.7, 148.5, 126.6, 125.1, 122.9, 115.1, 113.7, 113.2, 104.2, 99.2, 61.6, 56.3.

(*Z*)-2-(2,4-Dimethoxybenzylidene)-6-hydroxybenzofuran-3(2*H*)-one (2g) [1]

Compound **2g** was obtained from 6-hydroxy-3-coumaranone (300 mg, 2.0 mmol) and 2,4-dimethoxybenzaldehyde. Yield 97%.

^{1}H NMR (400 MHz, DMSO-d_6) δ 11.14 (brs, 1H), 8.13 (d, 1H, J = 8.8 Hz), 7.61 (d, 1H, J = 8.4 Hz), 7.02 (s, 1H), 6.78 (d, 1H), 6.72–6.67 (m, 3H), 3.91 (s, 3H), 3.85 (s, 3H).

(*Z*)-2-(2,5-Dimethoxybenzylidene)-6-hydroxybenzofuran-3(2*H*)-one (2h) [1]

Compound **2h** was obtained from 6-hydroxy-3-coumaranone and 2,5-dimethoxybenzaldehyde. Yield 93%.

^{1}H NMR (400 MHz, DMSO-d_6) δ 11.16 (brs, 1H), 7.70 (d, 1H, J = 2.4 Hz), 7.64 (d, 1H, J = 8.4 Hz), 7.05–7.02 (m, 3H), 6.82 (d, 1H, J = 1.8 Hz), 6.74 (dd, 1H, J = 8.4, 1.8 Hz), 3.85 (s, 3H), 3.79 (s, 3H); ^{13}C NMR (100 MHz, DMSO-d_6): δ 181.9, 168.4, 167.1, 153.6, 153.1, 148.0, 126.5, 121.4, 117.0, 116.7, 113.6, 113.3, 113.0, 104.1, 99.3, 56.7, 56.0.

(*Z*)-6-Hydroxy-2-(2,3,4-trimethoxybenzylidene)benzofuran-3(2*H*)-one (2i) [1]

Compound **2i** was obtained from 6-hydroxy-3-coumaranone and 2,3,4-trimethoxybenzaldehyde. Yield 95%.

^{1}H NMR (500 MHz, DMSO-d_6) δ 11.16 (brs, 1H), 7.90 (d, J = 8.4 Hz, 1H), 7.57 (d, J = 8.1 Hz, 1H), 6.96 (d, J = 6.9 Hz, 1H), 6.86 (s, 1H), 6.73 (d, J = 1.9 Hz, 1H), 6.67 (dd, J = 8.1, 1.9 Hz, 1H), 3.84 (s, 6H), 3.74 (s, 3H); ^{13}C NMR (125 MHz, DMSO-d_6): δ 181.6, 168.2, 166.9, 155.7, 153.5, 147.4, 142.2, 126.9, 126.5, 118.8, 113.5 (2C)p, 109.2, 104.6, 99.1, 62.2, 61.0, 56.6.

(*Z*)-6-Hydroxy-2-(3,4,5-trimethoxybenzylidene)benzofuran-3(2*H*)-one (2j) [1]

Compound **2j** was obtained from 6-hydroxy-3-coumaranone and 3,4,5-trimethoxybenzaldehyde. Yield 90%.

^{1}H NMR (400 MHz, DMSO-d_6) δ 11.21 (brs, 1H), 7.63 (d, 1H, J = 8.4 Hz), 7.32 (s, 2H), 6.83 (d, 1H, J = 2.0 Hz), 6.76 (s, 1H), 6.74 (dd, 1H, J = 8.4, 2.0 Hz), 3.86 (s, 6H), 3.74 (s, 3H).

1.1.2. General method General method for tandem O^6-alkylation of hispidol's analogs for preparation of compounds 3aa–3ak, 3am–3aq and 3at–3bd

To a solution of aurone derivatives **2a–2j** (0.66 mmol) and the appropriate aminoalkyl chloride hydrochloride salt (1.32 mmol) in acetone (30 mL) was added K_2CO_3 (700 mg, 5.0 mmol). The reaction mixture was refluxed until the reaction was completed from TLC monitoring. The mixture was concentrated in vacuo and partitioned between H_2O and CH_2Cl_2. The separated organic layer was washed with brine, dried over anhydrous Na_2SO_4, and filtered. The solvent was removed under reduced pressure and the residue was purified by silica gel column chromatography to give free amine form. To a solution of the free amine compound in EtOH was added acetyl chloride (370 mg, 4.7 mmol) at 0 °C. The reaction mixture was stirred in rt until the salt formation was completed.

The solvent was removed and the residue was precipitated from diethyl ether to afford the desired compounds.

(*Z*)-6-(2-(Diethylamino)ethoxy)-2-(2-methoxybenzylidene)benzofuran-3(2*H*)-one hydrochloride (3aa) [2]

Compound **3aa** was prepared from **2a** and 2-chloro-*N*,*N*-diethylethylamine hydrochloride according to general procedure 1.1.2. Yield 49%.

^{1}H NMR (400 MHz, CD_3OD) δ 8.15 (dd, *J*=1.6 Hz, *J*=6.4 Hz, 1H), 7.62 (d, *J*=8.8 Hz, 1H), 7.32 (ddd, *J*=6.4 Hz, *J*=1.8 Hz, *J*=1.2 Hz, 1H), 7.24 (1H, s), 6.99 (d, *J*=2.0 Hz, 1H), 6.94 (q, *J*=8.4 Hz, *J*=7.6 Hz, 2H), 6.83 (dd, *J*=6.4 Hz, *J*=2.0 Hz, 1H), 4.27 (t, *J*=4.8 Hz, 1H), 3.91 (s, 3H), 3.57 (t, *J*=4.8 Hz, 2H), 3.26 (q, *J*=7.6Hz, *J*=7.2 Hz, 4H), 1.29 (t, *J*=7.2Hz, 6H), ^{13}C NMR (100 MHz, CD_3OD) δ 184.6, 169.7, 167.5, 160.2, 148.9, 133.0, 132.9, 126.7, 122.0, 121.9, 113.9, 112.0, 111.5, 107.5, 98.8, 66.4, 66.3, 62.6, 56.2, 14.4; HRMS calcd for $C_{22}H_{26}NO_4$ $[M+H]^+$ 368.1862, found 368.1874.

(*Z*)-2-(2-Methoxybenzylidene)-6-(2-(pyrrolidin-1-yl)ethoxy)benzofuran-3(2*H*)-one hydrochloride (3ab) [1]

Compound **3ab** was prepared from **2a** and 1-(2-chloroethyl)pyrrolidine hydrochloride according to general procedure 1.1.2. Yield 25%.

^{1}H NMR (400 MHz, DMSO-d_6) δ 10.96 (brs, 1H), 8.20 (dd, 1H, *J* = 7.6, 1.2 Hz), 7.76 (d, 1H, *J* = 8.4 Hz), 7.49 (td, 1H, *J* = 7.6, 1.2 Hz), 7.27 (d, 1H, *J* = 2.0 Hz), 7.16–7.09 (m, 3H), 6.96 (dd, 1H, *J* = 8.4, 2.0 Hz), 4.56 (t, 2H, *J* = 4.8 Hz), 3.91 (s, 3H), 3.64 (t, 4H, *J* = 4.8 Hz), 1.96 (s, 4H); ^{13}C NMR (100 MHz, DMSO-d_6) δ 182.1, 168.2, 165.9, 158.7, 147.6, 132.3, 131.4, 126.1, 121.3, 120.6, 114.9, 113.6, 112.1, 105.0, 98.6, 64.8, 56.3, 54.1, 52.6, 23.1.

(*Z*)-2-(2-Methoxybenzylidene)-6-(3-(pyrrolidin-1-yl)propoxy)benzofuran-3(2*H*)-one hydrochloride (3ac) [1]

Compound **3ac** was prepared from **2a** and 1-(3-chloropropyl)pyrrolidine hydrochloride according to general procedure 1.1.2. Yield 76%.

^{1}H NMR (400 MHz, DMSO-d_6) δ 11.01 (brs, 1H), 8.17 (dd, 1H, *J* = 8.0, 1.6 Hz), 7.70 (d, 1H, *J* = 8.4 Hz), 7.46 (td, 1H, *J* = 8.0, 1.6 Hz), 7.15–7.07 (m, 4H), 6.86 (dd, 1H, *J* = 8.4, 2.0 Hz), 4.27 (t, 2H, *J* = 6.0 Hz), 3.89 (s, 3H), 3.53–3.52 (m, 2H), 3.28 (t, 2H, *J* = 8.0 Hz), 3.00–2.99 (m, 2H), 2.26–2.22 (m, 2H), 1.97–1.90 (m, 4H); ^{13}C NMR (100 MHz, DMSO-d_6) δ 181.6, 167.8, 166.2, 158.2, 147.2, 131.8, 130.9, 125.5, 120.8, 120.1, 114.0, 113.0, 111.6, 104.3, 97.7, 66.1, 55.8, 52.9, 50.9, 24.9, 22.7.

(*Z*)-6-(2-(Diethylamino)ethoxy)-2-(3-methoxybenzylidene)benzofuran-3(2*H*)-one hydrochloride (3ad) [2]

Compound **3ad** was prepared from **2b** and 2-chloro-*N*,*N*-diethylethanamine hydrochloride according to general procedure 1.1.2. Yield 34%.

^{1}H NMR (400 MHz, CD_3OD) δ 8.15 (dd, *J*=1.6 Hz, *J*=6.4 Hz, 1H), 7.62 (d, *J*=8.8 Hz, 1H), 7.32 (ddd, *J*=6.4 Hz, *J*=1.8 Hz, *J*=1.2 Hz, 1H), 7.24 (1H, s), 6.99 (d, *J*=2.0 Hz, 1H), 6.94 (q, *J*=8.4 Hz, *J*=7.6 Hz, 2H), 6.83 (dd, *J*=6.4 Hz, *J*=2.0 Hz, 1H), 4.27 (t, *J*=4.8 Hz, 1H), 3.91 (s, 3H), 3.57 (t, *J*=4.8 Hz, 2H), 3.26 (q, *J*=7.6Hz, *J*=7.2 Hz, 4H), 1.29 (t, *J*=7.2Hz, 6H), ^{13}C NMR (100 MHz, CD_3OD) δ 184.6, 169.7, 167.5, 160.2, 148.9, 133.0, 132.9, 126.7, 122.0, 121.9, 113.9, 112.0, 111.5, 107.5, 98.8, 66.4, 66.3, 62.6, 56.2, 14.4; HRMS calcd for $C_{22}H_{26}NO_4$ [M+H]$^+$ 368.1862, found 368.1872.

(*Z*)-6-(3-(diethylamino)propoxy)-2-(3-methoxybenzylidene)benzofuran-3(2*H*)-one hydrochloride (3ae) [2]

Compound **3ae** was prepared from **2b** and 1-(3-chloropropyl)diethylamine according to general procedure 1.1.2. Yield 46%.

^{1}H NMR (400 MHz, CD_3OD): δ 7.68 (d, *J* = 8.8 Hz, 2H), 7.48-7.46 (m, 2H), 7.37 (t, *J* = 8.0 Hz, 1H), 7.00-6.97 (m, 2H), 6.86 (dd, *J* = 8.8, 2.4 Hz, 1H) 6.76 (s, 1H), 4.28 (t, *J* = 5.6 Hz, 2H), 3.84 (s, 3H), 3.39-3.26 (m, 6H), 2.30-2.23 (m, 2H), 1.37 (t, *J* = 7.2 Hz, 6H); ^{13}C NMR (100 MHz, CD_3OD): δ 184.5, 170.1, 168.3, 161.5, 149.1, 134.7, 131.0, 126.8, 125.2, 117.6, 116.9, 115.9, 114.1, 113.4, 98.5, 67.1, 55.9, 50.3, 24.9, 9.3; HRMS calcd for $C_{23}H_{28}NO_4$ $[M+H]^+$ 382.2018, found 382.2031.

(*Z*)-2-(3-Methoxybenzylidene)-6-(2-(pyrrolidin-1-yl)ethoxy)benzofuran-3(2*H*)-one hydrochloride (3af) [1]

Compound **3af** was prepared from **2b** and 1-(2-chloroethyl)pyrrolidine hydrochloride according to general procedure 1.1.2. Yield 62%.

^{1}H NMR (400 MHz, DMSO-d_6) δ 11.03 (brs, 1H), 7.76 (d, 1H, *J* = 8.4 Hz), 7.58–7.57 (m, 2H), 7.45 (t, 1H, *J* = 8.4 Hz), 7.30 (d, 1H, *J* = 2.0 Hz), 7.05 (m, 1H), 6.96 (dd, 1H, *J* = 8.4, 2.0 Hz), 6.85 (s, 1H), 4.58 (t, 2H, *J* = 4.8 Hz), 3.83 (s, 3H), 3.63–3.62 (m, 4H), 3.12–3.10 (m, 2H), 1.99–1.91 (m, 4H); ^{13}C NMR (100 MHz, DMSO-d_6) δ 182.2, 168.3, 165.9, 159.9, 147.7, 133.6, 130.5, 126.1, 124.1, 117.0, 116.0, 114.8, 113.7, 111.7, 98.6, 64.9, 55.7, 54.3, 52.8, 23.0.

(*Z*)-2-(3-Methoxybenzylidene)-6-(3-(pyrrolidin-1-yl)propoxy)benzofuran-3(2*H*)-one hydrochloride (3ag) [1]

Compound **3ag** was prepared from **2b** and 1-(3-chloropropyl)pyrrolidine hydrochloride according to general procedure 1.1.2. Yield 59%.

^{1}H NMR (400 MHz, DMSO-d_6) δ 11.13 (brs, 1H), 7.71 (d, 1H, J = 8.4 Hz), 7.56–7.54 (m, 2H), 7.43 (t, 1H, J = 8.0 Hz), 7.19 (d, 1H, J = 2.0 Hz), 7.05 (dd, 1H, J = 8.0, 1.6 Hz), 6.87 (dd, 1H, J = 8.4, 2.0 Hz), 6.82 (s, 1H), 4.29 (t, 2H, J = 6.0 Hz), 3.82 (s, 3H), 3.54–3.53 (m, 2H), 3.29 (t, 2H, J = 8.0 Hz), 3.00 (brs, 2H), 2.28–2.22 (m, 2H), 1.98–1.90 (m, 4H); ^{13}C NMR (100 MHz, DMSO-d_6) δ 181.7, 168.0, 166.3, 159.4, 147.3, 133.2, 130.0, 125.5, 123.6, 116.4, 115.5, 113.9, 113.1, 110.9, 97.7, 66.2, 55.2, 52.9, 50.9, 24.9, 22.7.

(*Z*)-2-(3-Methoxybenzylidene)-6-(3-(piperidin-1-yl)propoxy)benzofuran-3(2*H*)-one hydrochloride (3ah) [1]

Compound **3ah** was prepared from **2b** and 1-(3-chloropropyl)pyrrolidine hydrochloride according to general procedure 1.1.2. Yield 47%.

^{1}H NMR (400 MHz, DMSO-d_6) δ 10.37 (brs, 1H), 7.73 (d, 1H, J = 8.4 Hz), 7.58–7.56 (m, 2H), 7.45 (t, 1H, J = 8.0 Hz), 7.21 (d, 1H, J = 1.6 Hz), 7.06 (dd, 1H, J = 8.0, 1.6 Hz), 6.88 (dd, 1H, J = 8.4, 1.6 Hz), 6.84 (s, 1H), 4.28 (t, 2H, J = 5.6 Hz), 3.83 (s, 3H), 3.47–3.15 (m, 4H), 2.93–2.87 (m, 2H), 2.27–2.24 (m, 2H), 1.80–1.37 (m, 6H); ^{13}C NMR (100 MHz, DMSO-d_6) δ 181.7, 168.0, 166.3, 159.5, 147.3, 133.2, 130.0, 125.6, 123.6, 116.4, 115.5, 113.9, 113.1, 111.0, 97.7, 66.3, 55.2, 53.1, 52.0, 23.0, 22.3, 21.4.

(*Z*)-2-(3-Methoxybenzylidene)-6-(2-morpholinoethoxy)benzofuran-3(2*H*)-one hydrochloride (3ai) [2]

Compound **3ai** was prepared from **2b** and 4-(2-chloroethyl)morpholine hydrochloride according to general procedure 1.1.2. Yield 48%.

^{1}H NMR (400 MHz, CD_3OD) δ 7.70 (d, J=8.4 Hz, 1H), 7.50 (t, J=2.4 Hz, J=9.2 Hz, 2H), 7.36 (t, J=7.8 Hz, 1H), 7.05 (d, J=2.0 Hz, 1H), 7.00 (dd, J=2.6 Hz, J=5.6 Hz, 1H), 6.89 (dd, J=2.0 Hz, J=6.8 Hz, 1H), 6.80 (s, 1H), 4.38 (t, J=5.2 Hz, 1H), 3.85 (s, 3H), 3.77 (t, J=4.8 Hz, 4H), 3.09 (s,

2H), 2.84 (s, 4H); ^{13}C NMR (100 MHz, CD_3OD) δ 184.5, 169.8, 167.1, 161.4, 149.0, 134.6, 130.9, 126.9, 125.2, 117.6, 116.9, 116.5, 114.0, 113.6, 99.0, 65.0, 64.0, 57.2, 55.8, 53.8, 18.3; HRMS calcd for $C_{22}H_{24}NO_5$ $[M+H]^+$ 382.1655, found 382.1668.

(*Z*)-2-(4-Methoxybenzylidene)-6-(2-(pyrrolidin-1-yl)ethoxy)benzofuran-3(2*H*)-one hydrochloride (3aj) [1]

Compound **3aj** was prepared from **2c** and 1-(2-chloroethyl)pyrrolidine hydrochloride according to general procedure 1.1.2. Yield 54%.

^{1}H NMR (400 MHz, DMSO-d_6) δ 10.91 (brs, 1H), 7.97 (d, 2H, J = 8.8 Hz), 7.75 (d, 1H, J = 8.8 Hz), 7.25 (s, 1H), 7.10 (d, 2H, J = 8.8 Hz), 6.95 (dd, 1H, J = 8.8, 2.0 Hz), 6.87 (s, 1H), 4.56 (t, 2H, J = 4.0 Hz), 3.85 (s, 3H), 3.63 (s, 4H), 3.13–3.11 (m, 2H), 1.99–1.92 (m, 4H); ^{13}C NMR (100 MHz, DMSO-d_6) δ 181.9, 167.9, 165.6, 161.1, 146.5, 133.6, 125.9, 124.9, 115.2, 115.1, 113.4, 112.1, 98.5, 64.7, 55.9, 54.0, 52.6, 23.1.

(*Z*)-2-(4-Methoxybenzylidene)-6-(3-(pyrrolidin-1-yl)propoxy)benzofuran-3(2*H*)-one hydrochloride (3ak) [1]

Compound **3ak** was prepared from **2c** and 1-(3-chloropropyl)pyrrolidine hydrochloride according to general procedure 1.1.2. Yield 85%.

^{1}H NMR (400 MHz, DMSO-d_6) δ 11.06 (brs, 1H), 7.96 (d, 2H, J = 8.8 Hz), 7.71 (d, 1H, J = 8.8 Hz), 7.16 (d, 1H, J = 2.0 Hz), 7.09 (d, 2H, J = 8.8 Hz), 6.87–6.85 (m, 2H), 4.29 (t, 2H, J = 6.0 Hz), 3.84 (s, 3H), 3.55 (brs, 2H), 3.31 (t, 2H, J = 7.6 Hz), 3.02 (brs, 2H), 2.30–2.24 (m, 2H), 1.99–1.92 (m, 4H); ^{13}C NMR (100 MHz, DMSO-d_6) δ 181.4, 167.6, 166.0, 160.6, 146.0, 133.1, 125.4, 124.5, 114.6, 114.3, 112.9, 111.5, 97.6, 66.1, 55.4, 52.9, 50.9, 24.9, 22.7.

(*Z*)-2-(4-Methoxybenzylidene)-6-(2-(piperidin-1-yl)ethoxy)benzofuran-3(2*H*)-one hydrochloride (3am) [1]

Compound **3am** was prepared from **2c** and 1-(2-chloroethyl)piperidine hydrochloride according to general procedure 1.1.2. Yield 43%.

^{1}H NMR (400 MHz, DMSO-d_6) δ 10.73 (brs, 1H), 7.97 (d, 2H, J = 8.8 Hz), 7.74 (d, 1H, J = 8.8 Hz), 7.25 (d, 1H, J = 1.6 Hz), 7.09 (d, 2H, J = 8.8 Hz), 6.93 (dd, 1H, J = 8.8, 1.6 Hz), 6.87 (s, 1H), 4.63 (t, 2H, J = 4.0 Hz), 3.84 (s, 3H), 3.52 (brs, 4H), 3.01–3.00 (m, 2H), 1.81–1.38 (m, 6H); ^{13}C NMR (100 MHz, DMSO-d_6): δ 182.8, 167.9, 165.3, 161.3, 146.2, 133.0, 125.1, 124.4, 115.1, 114.0, 112.8, 112.3, 97.2, 62.5, 55.2, 54.3, 53.3, 22.5, 21.0.

(*Z*)-2-(3,4-Dimethoxybenzylidene)-6-(2-(pyrrolidin-1-yl)ethoxy)benzofuran-3(2*H*)-one hydrochloride (3an) [1]

Compound **3an** was prepared from **2d** and 1-(2-chloroethyl)pyrrolidine hydrochloride according to general procedure 1.1.2. Yield 85%.

^{1}H NMR (400 MHz, DMSO-d_6) δ 11.13 (brs, 1H), 7.74 (d, 1H, J = 8.4 Hz), 7.67–7.57 (m, 2H) 7.26 (d, 1H, J = 1.8 Hz), 7.11 (d, 1H, J = 8.4 Hz), 6.95 (dd, 1H, J = 8.4, 1.8 Hz), 6.85 (s, 1H), 4.58 (t, 2H, J = 4.8 Hz), 3.86 (s, 3H), 3.84 (s, 3H), 3.64 (s, 4H), 3.12 (brs, 2H), 2.02–1.91 (m, 4H); ^{13}C NMR (100 MHz, DMSO-d_6) δ 181.9, 167.9, 165.6, 151.1, 149.3, 146.5, 125.9, 125.0, 115.2, 114.7, 113.4, 112.6, 112.4, 98.5, 64.8, 56.1, 54.1, 52.7, 23.1.

(*Z*)-2-(3,4-Dimethoxybenzylidene)-6-(3-(pyrrolidin-1-yl)propoxy)benzofuran-3(2*H*)-one hydrochloride (3ao) [1]

Compound **3ao** was prepared from **2d** and 1-(3-chloropropyl)pyrrolidine hydrochloride according to general procedure 1.1.2. Yield 66%.

^{1}H NMR (400 MHz, DMSO-d_6) δ 11.00 (brs, 1H), 7.71 (d, 1H, J = 8.8 Hz), 7.61–7.57 (m, 2H), 7.18 (d, 1H, J = 2.0 Hz), 7.11 (d, 1H, J = 8.4 Hz), 6.88 (dd, 1H, J = 8.8, 2.0 Hz), 6.84 (s, 1H), 4.30 (t, 2H, J = 6.0 Hz), 3.86 (s, 3H), 3.84 (s, 3H), 3.56 (brs, 2H), 3.31–3.29 (m, 2H), 3.01 (brs, 2H), 2.29–2.22 (m, 2H), 2.01–1.91 (m, 4H); ^{13}C NMR (100 MHz, DMSO-d_6) δ 181.4, 167.5, 166.0, 150.6, 148.8, 146.0, 125.3, 124.6, 114.3, 114.2, 112.9, 111.9, 97.7, 66.1, 64.9, 55.6, 52.9, 51.0, 24.9, 22.7.

(*Z*)-2-(3,4-Dimethoxybenzylidene)-6-(2-(piperidin-1-yl)ethoxy)benzofuran-3(2*H*)-one hydrochloride (3ap) [1]

Compound **3ap** was prepared from **2d** and 1-(2-chloroethyl)piperidine hydrochloride according to general procedure 1.1.2. Yield 65%.

^{1}H NMR (400 MHz, DMSO-d_6) δ 10.56 (brs, 1H), 7.74 (d, 1H, J = 8.4 Hz), 7.62–7.58 (m, 2H), 7.26 (d, 1H, J = 1.6 Hz), 7.11 (d, 1H, J = 8.4 Hz), 6.94 (dd, 1H, J = 8.4, 1.6 Hz), 6.86 (s, 1H), 4.61 (s, 2H), 3.86 (s, 3H), 3.84 (s, 3H), 3.53 (s, 4H), 3.03–3.01 (m, 2H), 1.81–1.38 (m, 6H); ^{13}C NMR (100 MHz, DMSO-d_6) δ 181.4, 167.4, 165.1, 150.7, 148.8, 146.0, 125.4, 124.6, 114.7, 114.3, 112.9, 112.1, 112.0, 98.1, 63.3, 55.6, 54.3, 52.6, 22.3, 21.2; HRMS m/z 410.19558 (calcd for $C_{24}H_{28}NO_5$ $[M-Cl]^+$, 410.19620).

(*Z*)-2-(3,4-Dimethoxybenzylidene)-6-(3-(piperidin-1-yl)propoxy)benzofuran-3(2*H*)-one hydrochloride (3aq) [1]

Compound **3aq** was prepared from **2d** and 1-(3-chloropropyl)pyrrolidine hydrochloride according to general procedure 1.1.2. Yield 94%.

^{1}H NMR (400 MHz, DMSO-d_6) δ 10.36 (s, 1H), 7.70 (d, 1H, J = 8.4 Hz), 7.61 (s, 1H), 7.58 (d, 1H, J = 8.4 Hz), 7.17 (d, 1H, J = 1.6 Hz), 7.09 (d, 1H, J = 8.4 Hz), 6.85 (dd, 1H, J = 8.4, 1.6 Hz), 6.84 (s, 1H), 4.28 (t, 2H, J = 5.9 Hz), 3.85 (s, 3H), 3.84 (s, 3H), 3.45 (brs, 2H), 3.18 (brs, 2H),

2.92–2.85 (m, 2H), 2.28–2.22 (m, 2H), 1.76–1.52 (m, 6H); ^{13}C NMR (100 MHz, DMSO-*d*$_6$) δ 184.2, 169.5, 167.9, 152.5, 150.5, 147.9, 127.3, 126.4, 116.0, 115.4, 114.3, 113.9, 112.7, 98.3, 67.1, 56.5, 56.4, 55.6, 25.0, 24.3, 22.7.

(*Z*)-2-(3,5-Dimethoxybenzylidene)-6-(2-(pyrrolidin-1-yl)ethoxy)benzofuran-3(2*H*)-one hydrochloride (3at) [1]

Compound **3at** was prepared from **2e** and 1-(2-chloroethyl)pyrrolidine hydrochloride according to general procedure 1.1.2. Yield 54%.

^{1}H NMR (400 MHz, DMSO-*d*$_6$) δ 11.16 (brs, 1H), 7.75 (d, 1H, *J* = 8.4 Hz), 7.31 (d, 1H, *J* = 2.0 Hz), 7.18 (d, 2H, *J* = 2.0 Hz), 6.96 (dd, 1H, *J* = 8.4, 2.0 Hz), 6.80 (s, 1H), 6.63 (t, 1H, *J* = 2.0 Hz), 4.63 (t, 2H, *J* = 4.8 Hz), 3.82 (s, 6H), 3.63–3.62 (m, 4H), 3.11 (brs, 2H), 2.01–1.91 (m, 4H); ^{13}C NMR (100 MHz, DMSO-*d*$_6$) δ 181.7, 167.8, 165.5, 160.6, 147.3, 133.5, 125.6, 114.3, 113.2, 111.2, 109.3, 101.9, 98.2, 64.4, 56.0, 55.4, 53.7, 52.2, 22.6.

(*Z*)-2-(3,5-Dimethoxybenzylidene)-6-(3-(pyrrolidin-1-yl)propoxy)benzofuran-3(2*H*)-one hydrochloride (3au) [1]

Compound **3au** was prepared from **2e** and 1-(3-chloropropyl)pyrrolidine hydrochloride according to general procedure 1.1.2. Yield 76%.

^{1}H NMR (400 MHz, DMSO-*d*$_6$) δ 11.03 (brs, 1H), 7.72 (d, 1H, *J* = 8.4 Hz), 7.22 (d, 1H, *J* = 1.6 Hz), 7.17 (d, 2H, *J* = 2.0 Hz), 6.88 (dd, 1H, *J* = 8.4, 1.6 Hz), 6.79 (s, 1H), 6.62 (t, 1H, *J* = 2.0 Hz), 4.30 (t, 2H, *J* = 6.0 Hz), 3.82 (s, 6H), 3.55–3.53 (m, 2H), 3.30 (t, 2H, *J* = 7.6 Hz), 3.02–3.00 (m, 2H), 2.28–2.24 (m, 2H), 1.96–1.91 (m, 4H); ^{13}C NMR (100 MHz, DMSO-*d*$_6$) δ 181.6, 168.0, 166.3, 160.6, 147.3, 133.5, 125.5, 113.9, 113.1, 112.0, 111.0, 109.2, 101.8, 97.8, 66.2, 58.1, 55.7, 55.3, 54.9, 52.9, 51.0, 24.9, 22.7.

(*Z*)-2-(2,3-Dimethoxybenzylidene)-6-(2-(pyrrolidin-1-yl)ethoxy)benzofuran-3(2*H*)-one hydrochloride (3av) [1]

Compound **3av** was prepared from **2f** and 1-(2-chloroethyl)pyrrolidine hydrochloride according to general procedure 1.1.2. Yield 43%.

^{1}H NMR (400 MHz, DMSO-d_6) δ 10.97 (brs, 1H), 7.82 (d, 1H, J = 7.6, 1.2 Hz), 7.77 (d, 1H, J = 8.4 Hz), 7.27–7.17 (m, 3H), 7.04 (s, 1H), 6.97 (dd, 1H, J = 8.4, 1.6 Hz), 4.57 (t, 2H, J = 4.6 Hz), 3.86 (s, 3H), 3.84 (s, 3H), 3.64 (t, 4H, J = 4.6 Hz), 3.17–3.12 (m, 2H), 1.97–1.92 (m, 4H); ^{13}C NMR (100 MHz, DMSO-d_6) δ 182.2, 168.2, 166.0, 153.0, 148.8, 148.2, 126.2, 125.9, 124.9, 122.8, 115.3, 114.8, 113.6, 104.9, 98.6, 64.8, 61.6, 56.3, 54.0, 52.6, 23.1.

(*Z*)-2-(2,4-Dimethoxybenzylidene)-6-(2-(pyrrolidin-1-yl)ethoxy)benzofuran-3(2*H*)-one hydrochloride (3aw) [1]

Compound **3aw** was prepared from **2g** and 1-(2-chloroethyl)pyrrolidine hydrochloride according to general procedure 1.1.2. Yield 56%.

^{1}H NMR (400 MHz, DMSO-d_6) δ 10.88 (brs, 1H), 8.16 (d, 1H, J = 8.4 Hz), 7.74 (d, 1H, J = 8.4 Hz), 7.24 (d, 1H, J = 1.6 Hz), 7.09 (s, 1H), 6.94 (dd, 1H, J = 8.4, 1.6 Hz), 6.72–6.69 (m, 2H), 4.55 (t, 2H, J = 4.8 Hz), 3.92 (s, 3H), 3.87 (s, 3H), 3.64–3.58 (m, 4H), 3.12 (brs, 2H), 1.96 (brs, 4H); ^{13}C NMR (100 MHz, DMSO-d_6) δ 181.8, 167.7, 165.5, 163.1, 160.5, 146.4, 132.7, 125.9, 115.3, 113.4, 107.3, 105.6, 98.7, 98.5, 64.8, 56.5, 56.1, 54.2, 52.8, 23.0.

(*Z*)-2-(2,5-Dimethoxybenzylidene)-6-(2-(pyrrolidin-1-yl)ethoxy)benzofuran-3(2*H*)-one hydrochloride (3ax) [1]

Compound **3ax** was prepared from **2h** and 1-(2-chloroethyl)pyrrolidine hydrochloride according to general procedure 1.1.2. Yield 89%.

^{1}H NMR (400 MHz, DMSO-d_6) δ 11.01 (brs, 1H), 7.74–7.72 (m, 2H), 7.30 (d, 1H, *J* = 1.6 Hz), 7.07–7.06 (m, 3H), 6.95 (dd, 1H, *J* = 8.4, 1.6 Hz), 4.56 (t, 2H, *J* = 4.8 Hz), 3.85 (s, 3H), 3.80 (s, 3H), 3.63–3.61 (m, 4H), 3.12–3.09 (m, 2H), 1.98–1.90 (m, 4H); ^{13}C NMR (100 MHz, DMSO-d_6) δ 182.1, 168.2, 165.9, 153.5, 153.2, 147.7, 126.1, 121.1, 117.2, 116.7, 114.9, 113.7, 113.0, 104.9, 98.7, 64.9, 56.7, 56.0, 54.1, 52.7, 23.1.

(*Z*)-2-(2,3-Dimethoxybenzylidene)-6-(3-(pyrrolidin-1-yl)propoxy)benzofuran-3(2*H*)-one hydrochloride (3ay) [1]

Compound **3ay** was prepared from **2f** and 1-(3-chloropropyl)pyrrolidine hydrochloride according to general procedure 1.1.2. Yield 68%.

^{1}H NMR (400 MHz, DMSO-d_6) δ 10.86 (brs, 1H), 7.81 (dd, 1H, *J* = 7.6, 1.2 Hz), 7.74 (d, 1H, *J* = 8.4 Hz), 7.25–7.17 (m, 3H), 7.03 (s, 1H), 6.89 (dd, 1H, *J* = 8.4, 2.0 Hz), 4.29 (t, 2H, *J* = 6.0 Hz), 3.86 (s, 3H), 3.84 (s, 3H), 3.55–3.54 (m, 2H), 3.30 (t, 2H, *J* = 7.6 Hz), 3.02–3.01 (m, 2H), 2.27–2.23 (m, 2H), 1.99–1.91 (m, 4H); ^{13}C NMR (100 MHz, DMSO-d_6) δ 181.7, 167.9, 166.3, 152.5, 148.3, 147.7, 125.6, 125.4, 124.4, 122.3, 114.8, 113.9, 113.1, 104.3, 97.7, 66.2, 61.1, 55.8, 52.9, 51.0, 24.9. 22.7.

(*Z*)-2-(2,4-Dimethoxybenzylidene)-6-(3-(pyrrolidin-1-yl)propoxy)benzofuran-3(2*H*)-one hydrochloride (3az) [1]

Compound **3az** was prepared from **2g** and 1-(3-chloropropyl)pyrrolidine hydrochloride according to general procedure 1.1.2. Yield 91%.

^{1}H NMR (400, DMSO-d_6) δ 11.00 (brs, 1H), 8.15 (d, 1H, *J* = 8.4 Hz), 7.70 (d, 1H, *J* = 8.8 Hz), 7.14 (s, 1H), 7.07 (s, 1H), 6.86 (d, 1H, *J* = 8.8 Hz), 6.71–6.69 (m, 2H), 4.28 (t, 2H, *J* = 6.0 Hz), 3.92 (s, 3H), 3.86 (s, 3H), 3.55–3.53 (m, 2H), 3.31–3.26 (m, 2H), 3.05–2.97 (m, 2H), 2.30–2.23 (m, 2H), 2.01–1.89 (m, 4H); ^{13}C NMR (100 MHz, DMSO-d_6) δ 181.3, 167.4, 165.9, 162.6, 159.9,

145.9, 132.2, 125.3, 114.3, 112.9, 112.8, 106.7, 104.9, 98.2, 97.6, 66.1, 56.0, 55.6, 55.1, 54.9, 54.4, 52.9, 52.8, 51.0, 24.9, 22.7.

(*Z*)-2-(2,5-Dimethoxybenzylidene)-6-(3-(pyrrolidin-1-yl)propoxy)benzofuran-3(2*H*)-one hydrochloride (3ba) [1]

Compound **3ba** was prepared from **2h** and 1-(3-chloropropyl)pyrrolidine hydrochloride according to general procedure 1.1.2. Yield 97%.

^{1}H NMR (400 MHz, DMSO-d_6) δ 10.86 (brs, 1H), 7.74–7.70 (m, 2H), 7.23 (d, 1H, J = 2.0 Hz), 7.07 (s, 3H), 6.88 (dd, 1H, J = 8.4, 2.0 Hz), 4.30 (t, 2H, J = 6.0 Hz), 3.86 (s, 3H), 3.80 (s, 3H), 3.55–3.53 (m, 2H), 3.30 (t, 2H, J = 7.6 Hz), 3.02–3.01 (m, 2H), 2.25 (p, 2H, J = 7.6, 6.0 Hz), 1.97–1.91 (m, 4H); ^{13}C NMR (100 MHz, DMSO-d_6) δ 181.5, 167.8, 166.3, 153.0, 152.7, 147.2, 125.5, 120.7, 116.7, 116.2, 114.0, 113.1, 112.5, 104.2, 97.8, 66.2, 56.2, 55.5, 54.9, 52.9, 51.0, 24.9, 22.7.

(*Z*)-6-(2-(Pyrrolidin-1-yl)ethoxy)-2-(2,3,4-trimethoxybenzylidene)benzofuran-3(2*H*)-one hydrochloride (3bb) [1]

Compound **3bb** was prepared from **2i** and 1-(2-chloroethyl)pyrrolidine hydrochloride according to general procedure 1.1.2. Yield 68%.

^{1}H NMR (400 MHz, DMSO-d_6) δ 11.07 (brs, 1H), 7.98 (d, 1H, J = 8.8 Hz), 7.76 (d, 1H, J = 8.4 Hz), 7.24 (d, 1H, J = 1.6 Hz), 7.01–6.93 (m, 3H), 4.57 (t, 2H, J = 4.8 Hz), 3.91 (s, 6H), 3.79 (s, 3H), 3.64–3.62 (m, 4H), 3.11 (brs, 2H), 1.97–1.91 (m, 4H); ^{13}C NMR (100 MHz, DMSO-d_6) δ 181.9, 167.9, 165.7, 155.8, 153.5, 147.1, 142.2, 126.9, 126.0, 118.6, 115.1, 113.4, 109.0, 105.4, 98.5, 64.7, 62.2, 60.9, 56.6, 54.0, 52.6, 23.1.

(*Z*)-6-(2-(Pyrrolidin-1-yl)ethoxy)-2-(3,4,5-trimethoxybenzylidene)benzofuran-3(2*H*)-one hydrochloride (3bc) [1]

Compound **3bc** was prepared from **2j** and 1-(2-chloroethyl)pyrrolidine hydrochloride according to general procedure 1.1.2. Yield 75%.

^{1}H NMR (400 MHz, DMSO-d_6) δ 11.17 (brs, 1H), 7.75 (d, 1H, J = 8.4 Hz), 7.35 (s, 2H), 7.28 (d, 1H, J = 1.8 Hz), 6.96 (dd, 1H, J = 8.4, 1.8 Hz), 6.84 (s, 1H), 4.59 (t, 2H, J = 4.8 Hz), 3.87 (s, 6H), 3.75 (s, 3H), 3.63 (s, 4H), 3.12 (brs, 2H), 1.92–1.91 (m, 4H); ^{13}C NMR (100 MHz, DMSO-d_6) δ 181.6, 167.6, 165.3, 153.0, 146.6, 139.4, 127.3, 125.5, 114.5, 113.1, 111.7, 109.0, 98.2, 64.4, 60.2, 56.1, 53.6, 52.2, 22.6.

(*Z*)-6-(2-(Piperidin-1-yl)ethoxy)-2-(3,4,5-trimethoxybenzylidene)benzofuran-3(2*H*)-one hydrochloride (3bd) [1]

Compound **3bd** was prepared from **2j** and 1-(2-chloroethyl)piperidine hydrochloride according to general procedure 1.1.2. Yield 80%.

^{1}H NMR (400 MHz, DMSO-d_6) δ 10.91 (brs, 1H), 7.75 (d, 1H, J = 8.4 Hz), 7.35 (s, 2H), 7.29 (d, 1H, J = 1.8 Hz), 6.94 (dd, 1H, J = 8.4, 1.8 Hz), 6.84 (s, 1H), 4.64 (t, 2H, J = 4.0 Hz), 3.88 (s, 6H), 3.75 (s, 3H), 3.51 (s, 4H), 3.02–3.01 (m, 2H), 1.82–1.38 (m, 6H); ^{13}C NMR (100 MHz, DMSO-d_6) δ 181.6, 167.6, 165.3, 153.0, 146.6, 139.4, 127.3, 125.5, 114.4, 113.0, 111.7, 109.0, 98.2, 63.4, 60.2, 56.1, 54.2, 52.5, 22.3, 21.2.

1.1.3. General method for tandem *O*^{6}-alkylation/*N*-alkylation of cyclic amines for preparation of compounds 3al, 3ar and 3as

To a solution of the appropriate 6-hydroxyaurone derivative (0.7 mmol) and the appropriate 1-bromo-chloroalkane (1.0 mmol) in DMF (10 mL) was added K_2CO and stirred at ambient temperature till consumption of the starting 6-hydroxyaurone derivative. To the *in situ* formed *O*^{6}-(chloroalkyl)-hispidol derivative the appropriate cycloalkylamine (1.2 mmol) and KI (186 mg, 1.12 mmol) were added and the reaction mixture was heated at 60°C for 9 hours, cooled to room

temperature, diluted with water and extracted with ethyl acetate. Drying of the organic extract ($NaSO_4$) followed by evaporation in vacuo and column chromatography afforded free amine form which was converted to the hydrochloride salt form as described under general procedure 1.1.2.

(*Z*)-2-(4-methoxybenzylidene)-6-(4-(pyrrolidin-1-yl)butoxy)benzofuran-3(2*H*)-one hydrochloride (3al)

Compound **3al** was prepared from **2c**, 1-bromo-4-chlorobutane, and pyrrolidine according to general procedure 1.1.3. Yield 68%.

^{1}H NMR (400 MHz, DMSO-d_6): δ 10.8 (brs, 1H), 7.96 (d, *J* = 8.8 Hz, 2H), 7.70 (d, *J* = 8.4 Hz, 1H), 7.15 (d, *J* = 2.0 Hz, 1H), 7.09 (d, *J* = 8.8 Hz, 2H), 6.87-6.84 (m, 2H), 4.19 (s, 2H), 3.84 (s, 3H), 3.51-3.50 (m, 2H), 3.17-2.97 (m, 6H), 1.85-1.84 (m, 6H); ^{13}C NMR (100 MHz, MeOD-d_4): δ 184.4, 169.8, 168.5, 162.8, 148.0, 137.3, 134.5, 126.5, 126.1, 118.4, 115.9, 115.6, 114.0, 98.3, 69.3, 58.7, 55.9, 55.2, 43.5, 27.1, 24.0, 22.7; HRMS calcd for $C_{24}H_{28}NO_4$ $[M+H]^+$ 394.2018, found 394.2028.

(*Z*)-2-(3,4-Dimethoxybenzylidene)-6-(4-(piperidin-1-yl)butoxy)benzofuran-3(2*H*)-one hydrochloride (3ar) [1]

Compound **3ar** was prepared from **2d**, 1-bromo-4-chlorobutane, and piperidine according to general procedure 1.1.3. Yield 55%.

^{1}H NMR (400 MHz, DMSO-d_6) *δ* 9.91 (s, 1H), 7.71 (d, 1H, *J* = 8.4 Hz), 7.61 (s, 1H), 7.58 (d, 1H, *J* = 8.4 Hz), 7.17 (d, 1H, *J* = 1.6 Hz), 7.09 (d, 1H, *J* = 8.4 Hz), 6.85 (dd, 1H, *J* = 8.4, 1.6 Hz), 6.84 (s, 1H), 4.28 (t, 2H, *J* = 5.9 Hz), 3.85 (s, 3H), 3.84 (s, 3H), 3.42 (brs, 2H), 3.08 (brs, 2H), 2.86–2.83 (m, 2H), 1.90–1.39 (m, 10H); ^{13}C NMR (100 MHz, DMSO-d_6) *δ* 184.2, 169.6, 168.4, 152.5, 150.5, 147.9, 127.2, 126.4, 115.7, 115.4, 114.1, 114.0, 112.7, 98.2, 69.3, 57.9, 56.6, 56.4, 54.3, 27.2, 24.3, 22.7, 22.0.

(*Z*)-2-(3,4-Dimethoxybenzylidene)-6-(5-(piperidin-1-yl)pentoxy)benzofuran-3(2*H*)-one hydrochloride (3as) [1]

Compound **3as** was prepared from **2d**, 1-bromo-5-chloropentane, and pyrrolidine according to general procedure 1.1.3. Yield 40%.

^{1}H NMR (400 MHz, DMSO-d_6) δ 9.91 (s, 1H), 7.68 (d, 1H, J = 8.4 Hz), 7.61 (d, 1H, J = 1.8 Hz), 7.58 (dd, 1H, J = 8.4, 1.8 Hz), 7.16 (d, 1H, J = 2.0 Hz), 7.10 (t, 1H, J = 8.4 Hz), 6.85 (dd, 1H, J = 8.4, 2.0 Hz), 6.83 (s, 1H), 4.19 (t, 2H, J = 6.3 Hz), 3.85 (s, 3H), 3.84 (s, 3H), 3.36 (brs, 2H), 3.00 (brs, 2H), 2.87–2.78 (m, 2H), 1.85–1.41 (m, 12H); ^{13}C NMR (100 MHz, DMSO-d_6) δ 182.8, 168.2, 167.2, 151.1, 149.1, 146.5, 125.8, 124.9, 114.2, 114.0, 112.7, 112.6, 111.3, 96.6, 68.3, 56.7, 55.1, 55.0, 52.9, 28.1, 23.3, 22.9, 22.8, 21.3.

2. Biological evaluations

2.1. *In vitro* assay of human monoamine oxidases inhibition

The MAO activity was quantified by measuring fluorescence intensity evoked by Amplex red reaction in which Amplex red reacts with hydrogen peroxide from the MAO reaction to produce highly fluorescent resorufin. The test compound in DMSO (final concentration: 1 nM – 10 μM or 10 nM – 100 μM) and hMAO enzyme (Sigma Aldrich, St. Louis, MO, USA) in 50 mM sodium phosphate buffer (pH 7.4, final protein amount: ~1.25 μg of MAO-A/well and 2.5 μg of MAO-B/well) were loaded on 96-well black plate and incubated for 15 min at 37 °C. Then working solution of 200 μM Amplex Red (Cayman, Ann Arbor, MI, USA), 1 U/mL horseradish peroxidase (Sigma-Aldrich) and 1 mM substrate (*p*-tyramine for MAO-A, benzylamine for MAO-B, Sigma-Aldrich) in 50 mM sodium phosphate buffer (pH 7.4) was added and incubated for 20 min at 37 °C in the dark. The fluorescence was measured using a microplate reader (SpectraMax® *i*3, Molecular Device, San Jose, CA, USA) with an excitation at 545 nm and an emission at 590 nm. Safinamide

and clorgyline were used as an positive control for MAO-B and MAO-A, respectively. The IC_{50} of compounds were determined as the mean ± S.E.M. in triplicate from the dose-response inhibition curves using SigmaPlot® 13.0.

2.2. *In vitro* acetylcholinesterase inhibition assay

Whole brains of male ICR mice (25–30 g) were homogenized in a glass Teflon homogenizer (Eyela, Japan) containing 50 volumes of phosphate buffer (pH 8.0, 0.1 M), and then centrifuged at 14,000 rpm for 20 min at 4 °C. The supernatant obtained was used as a source of enzyme for the assay. Each drug was initially dissolved in dimethyl sulfoxide (DMSO) and diluted to various concentrations immediately before use. An aliquot of diluted drug solution was then mixed with 640 μL of phosphate buffer (0.1 M, pH 8.0), 25 μL of buffered Ellman's reagent (10 mM 5,5′-dithio-bis(2-nitrobenzoic acid) and 15 mM sodium bicarbonate), and the enzyme source (100 μL) and pre-incubated at room temperature for 10 min. Then 5 μL of acetylthiocholine iodide solution (75 mM) was added to this mixture and mixed. Absorbance was measured at 410 nm after 10 min for the reaction adding 5 μL of acetylthiocholine iodide solution (75 mM) and to the reaction mixtures (OPTIZEN 2120UV, Mecasys Co. Ltd, Korea). The concentration of each drug required to inhibit acetylcholinesterase activity by 50% (IC_{50}) was calculated. Galantamine was used as a positive control.

2.3. *In vitro* cellular evaluations

2.3.1. Cell culture and viability determination

Murine BV2 microglial cells obtained from the Korean Cell Line Bank (Seoul, Republic of Korea) were cultured in DMEM with 10% FBS, 100 U/ml penicillin, and 100 μg/ml streptomycin at 37°C in a humidified 5% CO_2 atmosphere. Cell viability and toxicity were determined by the WST-1 assay (Roche, Mannheim, Germany). Briefly, BV2 cells in sterile plates were incubated with the

indicated concentrations of test compounds for 24 h. After replacing the culture media with fresh medium, WST-1 solution was added to each well and the cells were incubated for 2 h. Absorbance at 450 nm was then measured with a plate reader.

2.3.2. Inhibition of LPS-induced production of PGE_2

BV2 cells were incubated with various concentrations of each test compound for 2 h prior to incubation with or without LPS (500 ng/ml) for a further 22 h. The concentrations of PGE_2 in culture supernatants were assessed using enzyme immune assay kit (#ADI-900-001; Enzo Life Sciences, NY, USA) according to the manufacturer's instructions.

2.4. *In vivo* evaluations

2.4.1. Animals

Male CD-1 mice weighing 25-30 g were bought from Orient Co. Ltd, a branch of Charles River Laboratories (Seongnam-si, Gyeonggi-do, Korea) and kept in the Kyung Hee University Animal Care Unit for a week in groups of five per cage with unlimited access to food and water. The mice were housed under a constant humidity (60 ± 10%) and temperature (23 ± 1 °C) with 12 h light and dark cycles (illumination time 7:30-19:30). All the behavioral tests were conducted in light phase (11:00-16:00). Animal care and experimental procedures were conducted in accordance with the Animal Care and Use Guidelines issued by Kyung Hee University, Republic of Korea. The institutional animal care and use committee of Kyung Hee University approved the all experimental protocols using animals (approval No, KHSASP-22-022).

2.4.2. Evaluation of passive avoidance test

2.4.2.1. Drug administration

Mice were divided into 7 groups (n = 10) for the passive avoidance test at random. The groups were vehicle, vehicle with scopolamine (1 mg/kg, i.p.), scopolamine (1 mg/kg, i.p.) with compound **3aa** (1 or 10 mg/kg, p.o.), scopolamine (1 mg/kg, i.p.) with compound **3bc** (1 or 10 mg/kg, p.o.), and scopolamine (1 mg/kg, i.p.) with donepezil (5 mg/kg, p.o.). Compounds **3aa** and **3bc** were dissolved in distilled water. Scopolamine and donepezil were dissolved in 0.9 % saline solution. Mice were injected with scopolamine (1 mg/kg, i.p.) 30 min before the passive avoidance test. Compound **3aa** (1 or 10 mg/kg, p.o.), compound **3bc** (1 or 10 mg/kg, p.o.), donepezil (5 mg/kg, p.o.) or the same volume of vehicle was administered 1 h before passive avoidance test.

2.4.2.2. Passive avoidance task

The passive avoidance task, a fear-motivated test intended to assess capacity of mice for learning and memory, was carried out as previously reported [3]. The box for using the task was divided into two identical compartments (20 x 20 x 20 cm) that one of the boxes had 50 W bulb to illuminate and another had none, with a guillotine door (5 x 5 cm) between the two. The boxes had stainless steel rods (diameter: 2 mm) on the floor of the box. The experiment was required for 2 days, acquisition and retention trial days. Mice were placed gently in the corner of the light box for the acquisition trial and allowed to explore freely. After 30 s, the guillotine door was opened silently, and if mouse entered into the dark box, mouse was given an electric foot shock (0.5 mA, 3 s) through the grid. The retention trial was conducted 24 h after the acquisition trial. It was performed in the same way as the acquisition trial, but without electric foot shock. In each trial, the latency time for entering mice into the dark box was measured. The maximum latency for the acquisition trial and the retention trial was set at 60 s and 300 s, respectively.

2.4.3. Evaluation of forced swim test

2.4.4. Drug administration

Mice were divided into 6 groups (n = 10) for the forced swimming test at random. The groups were vehicle, compound 4aa (1 or 10 mg/kg), compound 4bc (1 or 10 mg/kg), and vehicle with desipramine (15 mg/kg). Compound 4aa and 4bc were dissolved in distilled water. Desipramine were dissolved in 0.9 % saline solution. Mice were administered compound 4aa (1 or 10 mg/kg, p.o.), compound 4bc (1 or 10 mg/kg, p.o.), or the same volume of vehicle was administered 1 h before forced swimming test. Desipramine (15 mg/kg, i.p.) were injected 30 min before the forced swimming test.

2.4.5. Forced swimming test

The forced swimming test was conducted, as described previously [4]. Mice were put into a glass cylinder sink (H x W: 25 x 14 cm), filled with 20 cm fresh water (24 ± 2 °C), and allowed to swim for 6 min. The swimming behaviors of each mouse were recorded by video camera. The feces of mice floating above the sink were fished out between each trial. The time of immobility, defined as few movements as necessary to keep its head above water, was analyzed during the last 4 min during 6 min using a video-based EthoVision System (Noldus, Wageningen, the Netherlands).

References

[1] Y.H. Lee, M.C. Shin, Y.D. Yun, S.Y. Shin, J.M. Kim, J.M. Seo, N.J. Kim, J.H. Ryu, Y.S. Lee, Synthesis of aminoalkyl-substituted aurone derivatives as acetylcholinesterase inhibitors, Bioorg. Med. Chem., 23 (2015) 231-240.
[2] A.H.E. Hassan, T.-N. Phan, S. Moon, C.H. Lee, Y.J. Kim, S.B. Cho, S.M. El-Sayed, Y. Choi, J.H. No, Y.S. Lee, Design, synthesis, and repurposing of O6-aminoalkyl-sulfuretin analogs towards discovery of potential lead compounds as antileishmanial agents, Eur. J. Med. Chem., 251 (2023) 115256.
[3] J. Kim, Y.H. Seo, J. Kim, N. Goo, Y. Jeong, H.J. Bae, S.Y. Jung, J. Lee, J.H. Ryu, Casticin ameliorates scopolamine-induced cognitive dysfunction in mice, Journal of ethnopharmacology, 259 (2020) 112843.
[4] J.M. Jung, S.J. Park, Y.W. Lee, H.E. Lee, S.I. Hong, J.H. Lew, E. Hong, J.S. Shim, J.H. Cheong, J.H. Ryu, The effects of a standardized Acanthopanax koreanum extract on stress-induced behavioral alterations in mice, Journal of ethnopharmacology, 148 (2013) 826-834.
